# CRISPR-Based Activation of Endogenous Expression of *TPM1* Inhibits Inflammatory Response of Primary Human Coronary Artery Endothelial and Smooth Muscle Cells Induced by Recombinant Human Tumor Necrosis Factor α

**DOI:** 10.3389/fcell.2021.668032

**Published:** 2021-09-17

**Authors:** Maciej Gagat, Wioletta Zielińska, Klaudia Mikołajczyk, Jan Zabrzyński, Adrian Krajewski, Anna Klimaszewska-Wiśniewska, Dariusz Grzanka, Alina Grzanka

**Affiliations:** ^1^Department of Histology and Embryology, Faculty of Medicine, Nicolaus Copernicus University in Toruń, Collegium Medicum in Bydgoszcz, Bydgoszcz, Poland; ^2^Department of Clinical Pathomorphology, Faculty of Medicine, Nicolaus Copernicus University in Toruń, Collegium Medicum in Bydgoszcz, Bydgoszcz, Poland; ^3^Department of General Orthopaedics, Musculoskeletal Oncology and Trauma Surgery, University of Medical Sciences, Poznań, Poland

**Keywords:** TNFα, endothelial cells, angiogenesis, F-actin, cell–cell junctions, cell adhesion, migrasomes, inflammation

## Abstract

Tumor necrosis factor α (TNFα) is one of the most important proinflammatory cytokines, which affects many processes associated with the growth and characteristics of endothelial, smooth muscle, and immune system cells. However, there is no correlation between most *in vivo* and *in vitro* studies on its role in endothelial cell proliferation and migration. In this study, we examined the effect of recombinant human (rh) TNFα produced in HEK293 cells on primary human coronary artery endothelial cells (pHCAECs) in the context of F-actin organization and such processes as migration and adhesion. Furthermore, we evaluated the possibility of the inhibition of the endothelial inflammatory response by the CRISPR-based regulation of *TPM1* gene expression. We showed that TNFα-induced activation of pHCAECs was related to the reorganization of the actin cytoskeleton into parallel-arranged stress fibers running along the longer axis of pHCAECs. It allowed for the directed and parallel motion of the cells during coordinated migration. This change in F-actin organization promoted strong but discontinuous cell–cell contacts involved in signalization between migrating cells. Moreover, this form of intercellular connections together with locally increased adhesion was related to the formation of migrasomes and further migracytosis. Stabilization of the actin cytoskeleton through the CRISPR-based activation of endogenous expression of *TPM1* resulted in the inhibition of the inflammatory response of pHCAECs following treatment with rh TNFα and stabilization of cell–cell junctions through reduced cleavage of vascular endothelial cadherin (VE-cadherin) and maintenance of the stable levels of α- and β-catenins. We also showed that CRISPR-based activation of *TPM1* reduced inflammatory activation, proliferation, and migration of primary human coronary artery smooth muscle cells. Therefore, products of the *TPM1* gene may be a potential therapeutic target for the treatment of proinflammatory vascular disorders.

## Introduction

The endothelial barrier plays a pivotal role in the regulation of the functioning of the entire circulatory system (Godo and Shimokawa, 2017). For this reason, any disturbances in the endothelial structure are associated with unfavorable health effects. Endothelial dysfunction manifests into abnormalities in the anticoagulant and anti-inflammatory properties of cells as well as alterations in vascular growth and vascular remodeling. It is associated with phenomena like hypertension and atherosclerosis (Chistiakov et al., 2015; Gimbrone and García-Cardeña, 2016). Endothelium produces nitric oxide, which is responsible for the relaxation of the underlying vascular smooth muscle. Reduced activity of endothelial nitric oxide synthase is observed in diabetes and hypertension. It also promotes vasoconstriction, thrombosis, and infiltration of the immune system cells (Matsushita et al., 2001). In turn, it is associated with the increased production of proinflammatory factors that induce the excessive proliferation of vascular smooth muscle cells. Proinflammatory cytokines also cause further accumulation of immune cells at the site of endothelial layer disruption. The increased production of endothelium-derived contracting factors leads to even greater deregulation of vessel wall functioning (Virdis et al., 2010).

Disbalance between the vasodilators and vasoconstrictors is not the only phenomenon leading to disruption of the endothelial barrier. From a biological perspective, endothelial dysfunction begins much earlier and manifests by intense changes in the structure, level, and function of membrane and intracellular proteins (Gimbrone and García-Cardeña, 2016). They are associated particularly with abnormalities at the level of junctional proteins, which can be observed as alterations in the pattern of intercellular connections. The types of cell contacts that guarantee the continuity of the endothelial layer are adherens junctions (AJs) and tight junctions (TJs). The main proteins involved in those types of connections in vascular endothelium are vascular endothelial cadherin (VE-cadherin) and claudin-5. However, proinflammatory conditions lead to a loosening of the junctional network, which manifests in the discontinuity at the cell–cell border and the appearance of spaces between cells (Cerutti and Ridley, 2017). These spaces allow, for example, massive infiltration of monocytes to the vessel wall, observed during the development and exacerbation of atherosclerosis. Other effects of proinflammatory conditions are enhanced cell adhesion and increased migration (Kaczmarek et al., 2005; Perna et al., 2013). In our work, we suggest that increased intracellular tensions result in the mechanistic disruption of intercellular junctions, which leads to the loss of the endothelial barrier continuity.

Endothelial injury, as well as angiogenesis, propels the migration of endothelial cells, which aims to restore the continuity of the endothelial monolayer or formation of new vessels (Michaelis, 2014). The cells can migrate both individually and in a coordinated manner depending on the intercellular signals and environmental factors. Collective migration is defined as the migration of cells that remain functionally and physically connected through stable intercellular connections. At the same time, such cells are characterized by multicellular polarity and the organization of the cytoskeleton that allows them to generate traction forces. In this type of migration, cell movement is coordinated locally and as a result, tightly connected cells move in the same direction (Canver et al., 2016).

All these changes, however, would not be possible without alterations at the actin cytoskeleton level. Changes in the organization of F-actin are involved in such processes as migration, proliferation, and cell adhesion. Moreover, the interaction of the actin cytoskeleton with junctional proteins allows the transfer of tensions from the relatively weak intercellular junctions to the more resistant cytoskeleton (van Geemen et al., 2014). In the case of non-activated endothelium, F-actin is organized into characteristic star-like structures that guarantee an appropriate distribution of intracellular tensions and enable a quick response to external factors, for example, through the ability of multidirectional migration. However, cell activation in response to proinflammatory cytokines involves the remodeling of the actin network into thick bundles of highly polymerized and parallel stress fibers. The changes in the organization of the actin cytoskeleton are possible only due to the activity of actin-binding proteins, which include tropomyosins (Gunning et al., 2015).

We have shown that tropomyosin-1 inhibits both polymerization and depolymerization of the actin filaments induced by L-homocysteine and cigarette smoke extract and impacts the integrity of EA.hy926 endothelial cells (Gagat et al., 2013, 2014). However, whether and how the expression of tropomyosin-1 and stabilization of actin cytoskeleton affect the inflammatory response of endothelial cells remain unknown. Therefore, the main goal of this study is to show that CRISPR-based activation of *TPM1* expression can maintain the proper organization of coronary artery endothelial cell monolayer and inhibit proliferation of smooth muscle cells in inflammatory conditions mimicked by the use of human recombinant (rh) tumor necrosis factor α (TNFα). In the present study, we characterized the effect of rh TNFα on primary human coronary artery endothelial cells (pHCAECs) in the context of F-actin organization and modulation of F-actin-dependent processes, including cell migration, adhesion, and migracytosis. The obtained results are important not only from the mechanistic point of view but also have the potential to be translated into clinical research and adapted during the design and development of new coronary stent devices.

## Materials and Methods

### Cell Culture and Treatment

Primary human coronary artery endothelial cells and primary human coronary artery smooth muscle cells (pHCASMCs) were obtained from a healthy 23-year-old white male or 53-year-old white male, respectively (cause of death: head trauma secondary to blunt injury). The cells were provided by the American Type Culture Collection (ATCC) and maintained according to supplier recommendations. Briefly, the endothelial cells were cultured in a vascular cell basal medium (ATCC) supplemented with an endothelial cell growth kit (ATCC) containing 5 ng/ml recombinant human (rh) VEGF, 5 ng/ml rh EGF, 5 ng/ml rh FGF basic, 15 ng/ml rh IGF-1, 10 mM L-glutamine, 0.75 units/ml heparin sulfate, 1 μg/ml hydrocortisone hemisuccinate, 2% fetal bovine serum (FBS), 50 μg/ml ascorbic acid, and antibiotics (10 units/ml penicillin, 10 μg/ml streptomycin, and 25 μg/ml amphotericin B). The smooth muscle cells were cultured in a vascular cell basal medium (ATCC) supplemented with a vascular smooth muscle cell growth kit (ATCC) containing 5 ng/ml rh FGF-basic, 5 μg/ml rh insulin, 50 μg/ml ascorbic acid, 10 mM L-glutamine, 5 ng/ml rh EGF, 5% fetal bovine serum, and antibiotics (10 units/ml penicillin, 10 μg/ml streptomycin, and 25 μg/ml amphotericin B). The cells were initially seeded at a density of 5,000 viable cells/cm^2^ and cultured in T-25 cm^2^ flasks (Corning) at 37°C in a humidified atmosphere of 5% CO_2_ and 95% air. After reaching confluence, the cells were treated with complete vascular growth medium containing rh TNFα (Sigma-Aldrich), expressed in HEK293 cells, at a concentration of 100 ng/ml for 24 h (or 36 h for time-dependent analysis of migration directiveness). The control cells were grown under the same condition without rh TNFα treatment. Only cells within three to four passages were used for all the experiments.

### Detection of Functional Expression of Adhesion Molecules

The detection of functional expression of E-selectin and VCAM-1 was performed using antibody-coated magnetic beads. The immobilization of Dynabeads M-280 Sheep Anti-Mouse IgG (Thermo Fisher Scientific) was done according to manufacturer instructions. Briefly, 2 μg of mouse anti-E-selectin (clone: CL2/6) or mouse anti-VCAM-1 (clone: 1.G11B1) monoclonal antibodies (both from Thermo Fisher Scientific) was added to 50 μl of pre-washed beads and resuspended in 1 ml of Ca^2+^- and Mg^2+^-free phosphate-buffered saline (PBS, pH 7.4), supplemented with 0.1% bovine serum albumin (BSA, Sigma-Aldrich) and 2 mM EDTA (Sigma-Aldrich), and incubated with gentle tilting and rotation for 18 h at 4°C. The beads were then washed four times using a washing buffer and resuspended in 1 ml of complete and pre-warmed vascular growth medium. Five hundred microliters of resuspended beads was added to 100 ng/ml TNFα-treated or control pHCAECs grown in 24-well glass-bottom cell imaging plates (Eppendorf) and incubated in standard cell culture conditions for 30 min. After washing with Dulbecco’s PBS (DPBS, ATCC), the cells were fixed in 4% paraformaldehyde in PBS for 20 min (Sigma-Aldrich) and stained for F-actin using Alexa Fluor 594 phalloidin (Thermo Fisher Scientific) for 20 min. The cells were then counterstained with 4′,6-diamidino-2-phenylindole (DAPI, Sigma-Aldrich) for 10 min. All steps were performed at room temperature (RT). Images were acquired on an Axio Observer Z1 inverted motorized microscope (Zeiss), using an EC Plan-Neofluar × 10/0.30 Ph1 air objective, Axiocam 503 mono camera, and ZEN 2 software (all from Zeiss). Parallel bright-field and fluorescence images were recorded at the same location.

### Co-culture of Primary Human Coronary Artery Endothelial Cells With Jurkat T Cells

Jurkat T cells (clone E6-1, ATCC) were maintained according to supplier recommendations. Briefly, the cells were initially seeded at a density of 1 × 10^5^ viable cells/ml and maintained in RPMI-1640 medium supplemented with 10% FBS and 50 μg/ml gentamycin (all from Lonza) in T-25 cm^2^ flasks (Corning) at 37°C in a humidified atmosphere of 5% CO_2_ and 95% air. After the desired density is reached, the cells were stimulated with 100 ng/ml rh TNFα for 2 h. The nuclei of cells were stained using the NucBlue Live Cell Stain ReadyProbes Reagent (Hoechst 33342 dye, Thermo Fisher Scientific) for 30 min. Around 2 × 10^5^ counterstained Jurkat T cells were then washed with DPBS, resuspended in 500 μl of complete and pre-warmed vascular growth medium and added to TNFα-activated or control pHCAECs grown in 12-well plates (Corning). Jurkat T cells were co-cultured with pHCAECs in standard cell culture conditions for 30 min in the presence or absence of rh TNFα. Before co-culture, pHCAECs were cultured in the presence or absence of rh TNFα for 24 h. Time-lapse images were acquired, at 3.33-s intervals over 2 min, in standard cell culture conditions on an Axio Observer Z1 inverted motorized microscope (Zeiss) equipped with an incubation system for live-cell imaging (PeCon), using an EC Plan-Neofluar × 10/0.30 Ph1 air objective, Axiocam 503 mono camera, and ZEN 2 software (all from Zeiss). Parallel contrast phase and fluorescence images were recorded at the same location. The number of adherent Jurkat T cells was counted after washing cells three times with DPBS in three randomly chosen microscopic fields using ImageJ (NIH).

### Confluent Cell Migration

Confluent cell migration assay was performed using six-well plates (Corning). After confluence is reached, the cells were cultured in complete vascular growth medium supplemented or not with 100 ng/ml rh TNFα. Time-lapse images were acquired, at 10-min intervals over 36 h in standard cell culture conditions on an Axio Observer Z1 inverted motorized microscope (Zeiss) equipped with an incubation system for live-cell imaging (PeCon), using an EC Plan-Neofluar × 10/0.30 Ph1 air objective, Axiocam 503 mono camera, and ZEN 2 software (all from Zeiss). Phase contrast images were recorded at a location of the migration gap and sites of open-field migration. Cell movement was analyzed using the Manual Tracking plugin for ImageJ (NIH). To quantify the dynamics of cell migration, such as velocity or Euclidean distance and accumulated distance, the migration trajectories were then assessed with Chemotaxis and Migration Tool 2.0 (Ibidi).

### Wound Healing and Open-Field Migration

Wound healing and open-field migration assays were performed using a two-well culture insert (Ibidi). pHCAECs were resuspended in 70 μl of complete vascular growth medium and plated at a density of 1,100 cells per well into each reservoir of a culture insert (Ibidi) that adhered to the bottom of a six-well plate (Corning) and grown in standard cell culture conditions. After confluence is reached, the insert was removed using forceps, and the plate was rinsed with DPBS to remove cell debris. Migrating cells were cultured in complete vascular growth medium supplemented or not with 100 ng/ml rh TNFα. Time-lapse images were acquired, at 10-min intervals over 24 h, in standard cell culture conditions on an Axio Observer Z1 inverted motorized microscope (Zeiss) equipped with an incubation system for live-cell imaging (PeCon), using an EC Plan-Neofluar × 10/0.30 Ph1 air objective, Axiocam 503 mono camera, and ZEN 2 software (all from Zeiss). Phase contrast images were recorded at a location of the migration gap and sites of open-field migration. Cell movement was analyzed using the Manual Tracking plugin for ImageJ (NIH). To quantify the dynamics of cell migration, such as velocity or Euclidean distance and accumulated distance, the migration trajectories were then assessed with Chemotaxis and Migration Tool 2.0 (Ibidi).

### Modified Boyden Chamber Assay

Modified Boyden chamber assay was done using uncoated Transwell inserts with microporous polyethylene terephthalate (PET) membranes (3-μm pore size) placed in 24-well plates (both from Corning). pHCAECs were resuspended at a density of 1,650 cells per well in complete vascular growth medium and plated in the upper chamber of the Transwell inserts. The lower chamber of the wells contained 600 μl of complete vascular growth medium. After confluence is reached, the media in the upper Transwell chamber were replaced with complete vascular growth medium supplemented or not with 100 ng/ml rh TNFα. The media in the wells of the 24-well plate were the same and additionally supplemented with 20% FBS. After 24 h, the Transwell inserts were fixed with 4% formaldehyde for 15 min (Sigma-Aldrich) and washed three times with PBS, and the cells were stained with toluidine blue. All steps were performed at RT. The cells on the upper side of the membrane were removed with cotton swabs, and the inserts were left to air-dry. Bright-field images of the lower side of the membranes were captured by an Eclipse E800 microscope (Nikon) using a Plan Fluor × 10/0.30 air objective, DS-5Mc-U1 camera, and NIS-Elements 3.30 (all from Nikon). The number of migrated cells was counted in randomly chosen microscopic fields using ImageJ (NIH).

### *In vitro* Tube Formation Assay

Endothelial tube formation assay was performed in μ-Slide Angiogenesis (Ibidi) according to the manufacturer’s guidelines. Briefly, inner wells were filled with 10 μl of ice-cold, growth factor-reduced, and phenol-red-free Matrigel Basement Membrane Matrix (Corning) and set at 37°C. pHCAECs were resuspended in 50 μl of complete vascular growth medium supplemented or not with 100 ng/ml rh TNFα and plated at a density of 10,000 cells per well into the upper well and cultured in standard conditions. Time-lapse images were acquired, at 10-min intervals over 24 h, in standard cell culture conditions on an Axio Observer Z1 inverted motorized microscope (Zeiss) equipped with an incubation system for live-cell imaging (PeCon), using a Zeiss EC Plan-Neofluar × 10/0.30 Ph1 air objective, Axiocam 503 mono camera, and ZEN 2 software (all from Zeiss). Contrast phase images were recorded at the same location. Additionally, tube formation was monitored using a bright-field microscope at 3, 6, 12, and 24 h after seeding. The cells were fixed with 4% formaldehyde for 15 min (Sigma-Aldrich), washed three times with PBS, and stained with crystal violet. All steps were performed at RT. Bright-field images were captured by an Eclipse E800 microscope (Nikon) using a Plan UW × 2/0.06 air objective, DS-5Mc-U1 camera, and NIS-Elements 3.30 (all from Nikon). The number of tubes was counted using ImageJ (NIH).

### Wash Assay

Wash assay was performed using six-well plates (Corning). The cells were initially seeded at a density of 5,000 viable cells/cm^2^ in complete vascular growth medium supplemented or not with 100 ng/ml rh TNFα and cultured in a humidified atmosphere of 5% CO_2_ and 95% air. At 0, 0.5, 2, and 4 h after seeding, the cells were washed three times with complete vascular growth to remove non-adherent cells. Contrast phase images were captured in standard cell culture conditions on an Axio Observer Z1 inverted motorized microscope (Zeiss) equipped with an incubation system for live-cell imaging (PeCon), using a Zeiss EC Plan-Neofluar × 10/0.30 Ph1 air objective, Axiocam 503 mono camera, and ZEN 2 software (all from Zeiss). The number of adherent cells was counted using ImageJ (NIH).

### Temporal Analysis of Actin Cytoskeleton and Focal Adhesion Sites

For temporal analysis of TNFα-induced changes in the organization of actin cytoskeleton and actin-mediated focal adhesion, pHCAECs were transiently transduced using a baculovirus system to express the green fluorescent protein (GFP)-fused human actin and the red fluorescent protein (RFP) fused to the c-terminus of human talin. The cells were transiently transduced with CellLight Reagents BacMam 2.0 (Thermo Fisher Scientific) according to the manufacturer’s recommendations. Briefly, pHCAECs were grown in 24-well cell imaging plates (Eppendorf) and cultured in standard conditions. After 70% confluence is reached, the cells were infected with CellLight actin-GFP and CellLight Talin-RFP BacMam 2.0 baculoviruses at a multiplicity of infection (MOI) of 30. After 16 h, the medium containing the baculoviruses was replaced with complete vascular growth medium supplemented or not with 100 ng/ml rh TNFα, and the cells were cultured in standard conditions. Time-lapse images were acquired, at 10-min intervals over 24 h, in standard cell culture conditions on an Axio Observer Z1 inverted motorized microscope (Zeiss) equipped with an incubation system for live-cell imaging (PeCon), using a Zeiss EC Plan-Neofluar × 10/0.30 Ph1 air objective, Axiocam 503 mono camera, and ZEN 2 software (all from Zeiss). Fluorescence images were recorded at the same location. Furthermore, correlation between 3D morphology and localization of actin-GFP and talin-RFP in rh TNF-activated pHCAECs was imaged using an HT-2 correlative holotomographic and fluorescence microscope (Tomocube). For live-cell correlative holotomographic and fluorescence imaging, the cells were grown in 50-mm imaging dishes with 1.5H glass coverslip bottom (TomoDish, Tomocube) and covered before imaging with a square coverslip glass. For prevention against contamination and drying of the medium, the side of the dish was sealed by mineral oil (Sigma). Additionally, expression of actin-GFP and talin-RFP was examined by confocal microscopy. The cells were fixed with 4% formaldehyde for 15 min (Sigma-Aldrich), washed three times with PBS, and counterstained with DAPI (Sigma-Aldrich) for 10 min. All steps were performed at RT. Images were captured by a C1 laser scanning confocal microscope (Nikon) using a Plan VC Apo × 60/1.4 oil objective and Nikon EZ-C1 3.80 software (both from Nikon). The lasers used for DAPI, GFP, and RFP excitations were diode 408 nm with emission filter 450/35, diode 488 nm with emission filter 515/30, and He–Ne 543 nm with emission filter 650LP, respectively. All confocal images of triple-labeled cells were acquired and displayed with identical settings, including laser power, pixel dwell speed, and gain.

### Coating With Extracellular Matrix Proteins

For the analysis of the possible effect of extracellular matrix (ECM) on different rearrangements of F-actin in TNFα-activated pHCAECs, the sterile glass coverslips (? 18 mm, Thermo Fisher Scientific) were covered by 5 g/cm^2^ fibronectin (source: human plasma), laminin (source: Engelbreth-Holm-Swarm mouse tumor), collagen I (source: rat tail tendon), and collagen IV (source: Engelbreth-Holm-Swarm mouse tumor; all from Corning) according the manufacturer’s instruction. The cells were grown on the coated glass coverslips in 12-well plates (Corning) and cultured in standard conditions. After confluence is reached, the cells were cultured for an additional period of 24 h in complete vascular growth medium supplemented or not with 100 ng/ml rh TNFα. The cells were then fixed with 4% formaldehyde for 15 min (Sigma-Aldrich), permeabilized for 10 min with 0.25% Triton X-100 (Serva), blocked with 3% BSA for 45 min (Sigma-Aldrich), and stained for F-actin using phalloidin–Alexa Fluor 488 (1:40) and phalloidin–Alexa Fluor 594 (1:40; both from Thermo Fisher Scientific). The cells were then counterstained with DAPI (Sigma-Aldrich) for 10 min. All of the fluorescence reaction steps were performed at RT. The slides were mounted in Aqua−Poly/Mount (Polysciences) and examined by confocal microscopy. Images were captured by a C1 laser scanning confocal microscope (Nikon) using a Plan VC Apo × 60/1.4 oil objective and Nikon EZ-C1 3.80 software (both from Nikon).

### Transfection by Nucleofection

Endogenous expression of *TPM1* gene was regulated using CRISPR *TPM1* activation or knockout systems (both from Santa Cruz). The CRISPR activation system is a synergistic activation mediator transcription activation system designed to specifically upregulate *TPM1* and consists of three plasmids at a 1:1:1 mass ratio: a plasmid encoding the deactivated Cas9 nuclease (D10A and N863A) fused to the transactivation domain VP64, a plasmid encoding the MS2-p65-HSF1 fusion protein, and a plasmid encoding a *TPM1*-specific 20-nt guide RNA. The CRISPR *TPM1*-knockout system consists of a pool of three plasmids each encoding the Cas9 nuclease and a *TPM1*-specific 20-nt guide RNA. As a CRISPR control, we used the CRISPR control plasmid encoding the Cas9 nuclease and a non-specific 20-nt guide RNA. Components of the above-mentioned CRISPR systems were delivered into the cells by nucleofection technology according to the manufacturer’s instruction using the P5 Primary Cell 4D-Nucleofector X Kit L and 4D-Nucleofector X unit (both from Lonza). Briefly, 4 × 10^5^ cells were resuspended in 100 Nucleofector nucleofection solution and 2 μg of each CRISPR system was electroporated using the DY138 program at RT. The cells were then immediately transferred into pre-warmed complete vascular growth medium and cultured until confluency is reached for further procedures.

### Fluorescence Localization of Proteins

For the fluorescence localization of proteins, the pHCAECs were grown on sterile glass coverslips (? 18 mm, Thermo Fisher Scientific) in 12-well plates (Corning) and cultured in standard conditions. After confluence is reached, the cells were cultured for an additional period of 24 h in complete vascular growth medium supplemented or not with 100 ng/ml rh TNFα. The cells were then fixed with 4% formaldehyde for 15 min (Sigma-Aldrich), permeabilized for 10 min with 0.25% Triton X-100 (Serva), and blocked with 3% BSA for 45 min (Sigma-Aldrich). Next, the cells were double stained using antibodies and phalloidin conjugates in the following arrangement: (i) mouse anti-talin (clone: 8D4) monoclonal antibody (1:500, Sigma-Aldrich), goat anti-mouse–Alexa Fluor 488 secondary antibody (1:200, Thermo Fisher Scientific), and phalloidin–Alexa Fluor 594 (1:40, Thermo Fisher Scientific); (ii) rabbit anti-VE-cadherin polyclonal antibody (1:200), donkey anti-rabbit–Alexa Fluor 594 secondary antibody (1:200), and phalloidin–Alexa Fluor 488 (1:40; all from Thermo Fisher Scientific); (iii) mouse anti-α-catenin (clone: 7A4) monoclonal antibody (1:250), goat anti-mouse–Alexa Fluor 488 secondary antibody (1:200), and phalloidin–Alexa Fluor 594 (1:40; all from Thermo Fisher Scientific); (iv) mouse anti-β-catenin (clone: 5H10) monoclonal antibody (1:250), goat anti-mouse–Alexa Fluor 488 secondary antibody (1:200), and phalloidin–Alexa Fluor 594 (1:40; all from Thermo Fisher Scientific); (v) mouse anti-ZO-1 (clone: 1A12) monoclonal antibody (1:200), goat anti-mouse–Alexa Fluor 488 secondary antibody (1:200), and phalloidin–Alexa Fluor 594 (1:40; all from Thermo Fisher Scientific); (vi) mouse anti-claudin-5 (clone: 4C3C2) monoclonal antibody (1:300), goat anti-mouse–Alexa Fluor 488 secondary antibody (1:200), and phalloidin–Alexa Fluor 594 (1:40; all from Thermo Fisher Scientific); (vii) rabbit anti-non-muscle myosin IIa (MYH9) polyclonal antibody (1:100), donkey anti-rabbit–Alexa Fluor 594 secondary antibody (1:200), and phalloidin–Alexa Fluor 488 (1:40; all from Thermo Fisher Scientific); (viii) rabbit anti-non-muscle myosin IIb (MYH10) polyclonal antibody (1:100), donkey anti-rabbit–Alexa Fluor 594 secondary antibody (1:200), and phalloidin–Alexa Fluor 488 (1:40; all from Thermo Fisher Scientific); (ix) rabbit anti-Arp 2/3 subunit 1B (ARPC1B) polyclonal antibody (1:100, Abcam), donkey anti-rabbit-Alexa Fluor 594 secondary antibody (1:200, Thermo Fisher Scientific), and phalloidin–Alexa Fluor 488 (1:40, Thermo Fisher Scientific); (x) rabbit anti-ROCK-1 polyclonal antibody (1:100), donkey anti-rabbit–Alexa Fluor 594 secondary antibody (1:200), and phalloidin–Alexa Fluor 488 (1:40; all from Thermo Fisher Scientific); (xi) rabbit anti-ROCK-2 polyclonal antibody (1:100), donkey anti-rabbit–Alexa Fluor 594 secondary antibody (1:200), and phalloidin–Alexa Fluor 488 (1:40; all from Thermo Fisher Scientific); (xii) mouse anti-E-selectin (clone: CL2/6) monoclonal antibody (1:100), goat anti-mouse–Alexa Fluor 488 secondary antibody (1:200), and phalloidin–Alexa Fluor 594 (1:40; all from Thermo Fisher Scientific); and (xiii) mouse anti-VCAM-1 (clone: 1.G11B1) monoclonal antibody (1:200), goat anti-mouse–Alexa Fluor 488 secondary antibody (1:200), and phalloidin–Alexa Fluor 594 (1:40; all from Thermo Fisher Scientific). The pHCASMCs were grown in similar conditions and stained using (i) mouse anti-ICAM-1 (clone: 1A29) monoclonal antibody (1:250) and goat anti-mouse–Alexa Fluor 594 secondary antibody (1:200; both from Thermo Fisher Scientific); (ii) mouse anti-VCAM-1 (clone: 1.G11B1) monoclonal antibody (1:200) and goat anti-mouse–Alexa Fluor 488 secondary antibody (1:200; both from Thermo Fisher Scientific); (iii) rabbit anti-CDKN2A/p16INK4a (clone: EPR1473) monoclonal antibody (1:100, Abcam) and donkey anti-rabbit–Alexa Fluor 594 secondary antibody (1:200, Thermo Fisher Scientific); (iv) rabbit anti-NFκB p65 polyclonal antibody (1:200) and donkey anti-rabbit–Alexa Fluor 594 secondary antibody (1:200; both from Thermo Fisher Scientific); and (v) rabbit CCN4/WISP1 polyclonal antibody (1:500) and donkey anti-rabbit–Alexa Fluor 594 secondary antibody (1:200; both from Thermo Fisher Scientific). All incubations with antibodies and/or phalloidin conjugates were carried out, respectively, for 1 h or 20 min, followed by three washes in PBS. The cells were then counterstained with DAPI (Sigma-Aldrich) for 10 min. All steps were performed at RT. The slides were mounted in Aqua−Poly/Mount (Polysciences) and examined by confocal microscopy. Images were captured by a C1 laser scanning confocal microscope (Nikon) using a Plan VC Apo × 60/1.4 oil objective and Nikon EZ-C1 3.80 software (both from Nikon). The lasers used for DAPI, Alexa Fluor 488, and Alexa Fluor 596 excitations were diode 408 nm with emission filter 450/35, diode 488 nm with emission filter 515/30, and He–Ne 543 nm with emission filter 650LP, respectively. All confocal images of triple-labeled cells were acquired and displayed with identical settings, including laser power, pixel dwell speed, and gain. The gain was set up for optimal visibility of specific proteins. In the case of the lack of possibility to show triple-labeled cells, the gain of the channel for F-actin was downregulated. The fluorescence intensity of selected proteins was quantified using ImageJ (NIH) and EZ-C1 (Nikon).

### Immunoblot

Control pHCAECs and those treated with 100 ng/ml TNFα were lysed with RIPA buffer (Sigma-Aldrich) supplemented with Halt protease inhibitor cocktail (Thermo Fisher Scientific). After clarification of lysates (8,000 × *g* for 10 min at 4°C) and normalization of protein concentration by a BCA protein assay kit, 10 μg of total protein per lane was separated by SDS-PAGE at 225 V with Novex WedgeWell 10%–20% Tris-glycine gel and Mini Gel Tank (all from Thermo Fisher Scientific). The transfer onto a nitrocellulose membrane was performed by iBlot Dry Western Blotting System (Life Technologies). The membrane was then blocked for 10 min in a SuperSignal Western Blot Enhancer (Thermo Fisher Scientific) and incubated for 2.5 h or overnight in an iBind Flex Solution (Thermo Fisher Scientific) with (i) mouse anti-claudin-5 (clone: 4C3C2) monoclonal antibody (1:500) and goat anti-mouse–horseradish peroxidase secondary antibody (HRP, 1:2,000; both from Thermo Fisher Scientific); (ii) mouse anti-GAPDH (clone: ZG003) monoclonal antibody (1:500) and goat anti-mouse–HRP secondary antibody (1:2,000; both from Thermo Fisher Scientific); (iii) mouse anti-α-tropomyosin (clone: F-6) monoclonal antibody (1:100, Santa Cruz) and goat anti-mouse-HRP secondary antibody (1:2,000, Thermo Fisher Scientific); (iv) mouse anti-E-selectin (clone: CL2/6) monoclonal antibody (1:100) and goat anti-mouse–HRP secondary antibody (1:2,000; both from Thermo Fisher Scientific); (v) mouse anti-talin (clone: 8D4) monoclonal antibody (1:100, Sigma-Aldrich) and goat anti-mouse–HRP secondary antibody (1:2,000, Thermo Fisher Scientific); (vi) mouse anti-vinculin (clone: J144) monoclonal antibody (1:1,000) and goat anti-mouse-HRP secondary antibody (1:2,000; both from Thermo Fisher Scientific); (vii) mouse anti-α-catenin (clone: 7A4) monoclonal antibody (1:250) and goat anti-mouse–HRP secondary antibody (1:2,000; both from Thermo Fisher Scientific); (viii) mouse anti-β-catenin (clone: 5H10) monoclonal antibody (1:250) and goat anti-mouse–HRP secondary antibody (1:2,000; both from Thermo Fisher Scientific); (iv) rabbit anti-VE-cadherin polyclonal antibody (1:500) and goat anti-mouse–HRP secondary antibody (1:2,000; both from Thermo Fisher Scientific) using the iBind Flex Western System (Thermo Fisher Scientific). Protein bands were visualized using the 1-Step Ultra TMB-Blotting Solution (Thermo Fisher Scientific). All steps were performed at RT. All western blot assays were performed at least three times. Images were captured by the ChemiDoc MP Imaging System (Bio-Rad). Intensity of bands was quantified using ImageJ (NIH).

### Statistical Analysis

The significant differences between two groups were calculated using an unpaired *t*-test or one-way ANOVA and Kruskal–Wallis test with Dunn’s correction for multiple comparisons. Relations between cell migration parameters were assessed using Pearson’s correlation coefficient analysis. Statistical analyses were performed by using Prism 7 software (GraphPad). The Rayleigh test was used to determine cell movement homogeneity using Chemotaxis and Migration Tool 2.0 (Ibidi). A *p*-value < 0.05 was considered to be statistically significant and labeled on figures as ^∗^ or $, *p* < 0.05; ^∗∗^ or $$, *p* < 0.01; ^∗∗∗^ or $$$, *p* < 0.001; ^****^ or $$$$, *p* < 0.0001; and NS, non-significant. Results were expressed as means ± SD.

## Results

### Tumor Necrosis Factor α Induces Inflammatory Activation of Primary Human Coronary Artery Endothelial Cells

**FIGURE 1:**
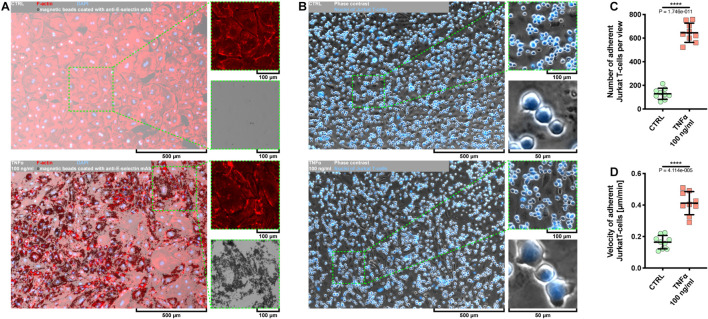
Effect of TNFα on activation of pHCAECs. **(A)** Representative micrographs of intravital detection of E-selectin using magnetic beads coated with anti-E-selectin monoclonal antibodies (bright field). F-actin (red) and nuclei (blue) were stained fluorescently after fixation. CTRL refers to the control (top panel), TNFα 100 ng/ml to cells treated with rh TNFα (bottom panel), mAb to monoclonal antibodies. **(B)** Representative micrographs of intravital co-culture of pHCAECs (contrast phase) with Jurkat T cells (contrast phase and fluorescence). Nuclei of Jurkat T cells (blue) were stained with Hoechst 33342. CTRL refers to the control (top panel), TNFα 100 ng/ml to cells treated with rh TNFα (bottom panel). **(C)** Number of adherent Jurkat T cells to pHCAECs per microscopic field from the control and TNFα-activated pHCAECs after 30 min of their co-culture. CTRL refers to the control (top panel), TNFα 100 ng/ml to cells treated with rh TNFα, **** to *p* < 0.0001, as determined by unpaired *t*-test. **(D)** Velocity of motile Jurkat T cells on pHCAECs during 30 min of their co-culture. CTRL refers to the control (top panel), TNFα 100 ng/ml to cells treated with rh TNFα. ****p < 0.0001, as determined by unpaired *t*-test.

Given that the use of *Escherichia coli* as an expression platform for recombinant proteins has several drawbacks (Rosano and Ceccarelli, 2014), we first examined whether rh TNFα expressed in HEK293 cells was able to activate coronary artery endothelial cells. To this end, pHCAECs were cultured to 100% confluence and then treated for 24 h with 100 ng/ml rh TNFα. We showed rh TNFα-induced surface and intracellular expressions of functional E-selectin (Figure 1A and Supplementary Figure S1) and VCAM-1 (Supplementary Figures S2A,B). We then examined the interactions between rh TNFα-activated pHCAECs and Jurkat T cells (Figure 1B and Supplementary Video S1). We observed a statistically significant increase in the number of Jurkat T cells adherent to pHCAECs (from 128.6 ± 47.00 per view in the control to 645 ± 81.01 in TNFα-activated cells, *p* < 0.0001) after 30 min of their co-culture (Figure 1C and Supplementary Figure S2C). Furthermore, we showed a statistically significant increase in the velocity of motile Jurkat T cells on pHCAECs (from 0.1641 ± 0.04247 to 0.4120 ± 0.07306 μm/min, *p* < 0.0001, respectively, for control and TNFα-activated pHCAECs; Figure 1D).

In summary, these data suggest that rh TNFα effectively activates pHCAECs and augments flattening, firm adhesion, and further migration of T cells on the endothelial monolayer.

### Tumor Necrosis Factor α Augments the Invasive Potential of Primary Human Coronary Artery Endothelial Cells

**FIGURE 2:**
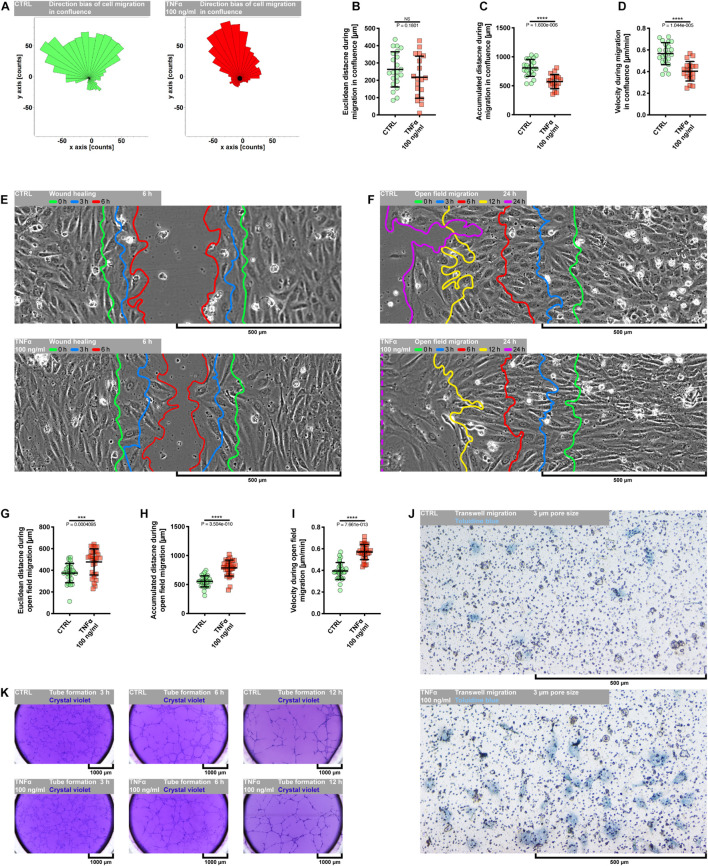
Effect of TNFα on migration of pHCAECs. **(A)** Rose plot presenting direction bias of pHCAEC migration in confluence within 24 h. CTRL refers to the control (left panel), TNFα 100 ng/ml to cells treated with rh TNFα (right panel), ** to *p* < 0.01, **** to *p* < 0.0001, as determined by Rayleigh test. **(B)** Euclidean distance of pHCAECs during 24-h migration in 100% confluence. CTRL refers to the control, TNFα 100 ng/ml to cells treated with rh TNFα, NS to non-significant to the control, as determined by unpaired *t*-test. **(C)** Accumulated distance of pHCAECs during 24-h migration in 100% confluence. CTRL refers to the control, TNFα 100 ng/ml to cells treated with rh TNFα, **** to *p* < 0.0001, as determined by unpaired *t*-test. **(D)** Velocity of pHCAECs during 24-h migration in 100% confluence. CTRL refers to the control, TNFα 100 ng/ml to cells treated with rh TNFα, **** to *p* < 0.0001, as determined by unpaired *t*-test. **(E)** Representative contrast phase micrographs presenting pHCAEC migration to the wound site. Curves indicate area without cells in 0 h (green), 3 h (blue), and 6 h (red) of the wound healing assay. CTRL refers to the control (upper panel), TNFα 100 ng/ml to cells treated with rh TNFα (lower panel). **(F)** Representative contrast phase micrographs presenting pHCAEC migration to the open field. Curves indicate area without cells in 0 h (green), 3 h (blue), 6 h (red), 12 h (yellow), and 24 h (purple) of the open-field migration assay. CTRL refers to the control (upper panel), TNFα 100 ng/ml to cells treated with rh TNFα (lower panel). **(G)** Euclidean distance of pHCAECs during 24-h open-field migration. CTRL refers to the control, TNFα 100 ng/ml to cells treated with rh TNFα, *** to *p* < 0.001, as determined by unpaired *t*-test. **(H)** Accumulated distance of pHCAECs during 24-h open-field migration. CTRL refers to the control, TNFα 100 ng/ml to cells treated with rh TNFα, **** to *p* < 0.0001, as determined by unpaired *t*-test. **(I)** Velocity of pHCAECs during 24-h open-field migration. CTRL refers to the control, TNFα 100 ng/ml to cells treated with rh TNFα, **** to *p* < 0.0001, as determined by unpaired *t*-test. **(J)** Representative bright-field micrographs presenting migration of pHCAECs through 3-μm pores. The cells were stained with toluidine blue. CTRL refers to the control (upper panel), TNFα 100 ng/ml to cells treated with rh TNFα (lower panel). **(K)** Representative bright-field micrographs presenting tube formation by pHCAECs cultured on Matrigel. The tubular structures were stained with crystal violet after 3 h (left panel), 6 h (middle panel), and 12 h (right panel) from seeding. CTRL refers to the control (upper panels), TNFα 100 ng/ml to cells treated with rh TNFα (lower panels).

Evidence from *in vitro* and *in vivo* data has demonstrated a dual role of TNFα in the angiogenic response of endothelial cells. It has been shown that TNFα generally exerts a proangiogenic effect *in vivo* and antiangiogenic *in vitro* (Fràter-Schröder et al., 1987; Sainson et al., 2008). However, it seems to function as a stimulatory or inhibitory agent dependent on the model used and experimental condition. As depicted in Supplementary Figures S1, S2B, the exposure of pHCAECs to 100 ng/ml rh TNFα resulted in remarkable changes in the cell morphology, whereby cells appeared enlarged and elongated. We next examined whether these morphological changes were accompanied by enhanced migration capacity of pHCAECs and ability to form tubules on the Matrigel. We first investigated the migration pattern of pHCAECs cultured in the confluence. As shown on rose plots in Figure 2A, during 24 h of incubation with 100 ng/ml rh TNFα, pHCAECs moved in a more persistent manner, with decreased changes in direction, whereas control cells exhibited a more random migration pattern, suggesting that TNFα promoted directional migration of pHCAECs. This finding was further supported by a high significance of the Rayleigh test (from *p* = 2.74694 × 10^–3^ to *p* = 4.90393 × 10^–5^, respectively, for control and TNFα-activated cells). As cultured in 100% confluence for 24 h, there was no effect of rh TNFα on Euclidean distance of migrating cells (Figure 2B). However, the accumulated distance traveled by rh TNFα-activated pHCAECs was significantly shorter compared to the control cells (from 808.8 ± 146.2 to 573.5 ± 124.0 μm, *p* < 0.0001; Figure 2C). Furthermore, we showed a statistically significant decrease in the velocity of these cells (from 0.5656 ± 0.1023 to 0.4035 ± 0.09019 μm/min, *p* < 0.0001, respectively, for control and TNFα-activated cells; Figure 2D). The extended tracking time of migrating cells for up to 36 h allowed us to demonstrate that longer rh TNFα treatment also promoted the migration of pHCAECs in a more directional manner (Rayleigh test: *p* = 2.96697 × 10^–2^ *vs. p* = 4.24461 × 10^–5^; Supplementary Figure S3A) without the effect on Euclidean distance (Supplementary Figure S3B). Longer exposition of pHCAECs to rh TNFα also revealed shorter accumulated distance of migrating cells (1215.0 ± 194.4 *vs.* 816.7 ± 194.6, *p* < 0.0001; Supplementary Figure S3C) related with their decreased velocity (0.5626 ± 0.09002 *vs.* 0.3781 ± 0.09008 μm/min, *p* < 0.0001; Supplementary Figure S3D), when compared to untreated pHCAECs. Furthermore, there was a negative correlation between time of treatment and Euclidean distance (*r* = −0.5166, *p* = 0.0013), accumulated distance (*r* = −0.4916, *p* = 0.0023), and velocity (*r* = −0.4837, *p* = 0.0028; Supplementary Figures S3E–G). Additional analysis revealed that Euclidean distance (*r* = 0.6206, *p* < 0.0001), accumulated distance (*r* = 0.3723, *p* = 0.0253), and velocity (*r* = 0.3790, *p* = 0.0226), but not time of treatment, were correlated with the directness of rh TNFα-activated pHCAECs (Supplementary Figure S3I), giving further support for the conclusion that pHCAECs undergo directed migration upon rh TNFα treatment. In control cells, we only observed the correlation between directness of cells and Euclidean distance (*r* = 0.7929, *p* < 0.0001; Supplementary Figure S3H).

Differently, wound healing (Figure 2E and Supplementary Video S2) and open-field migration (Figure 2F and Supplementary Video S3) assays showed faster migration of rh TNFα-activated pHCAECs as compared to the control, especially from the third hour of wound healing experiment or the 12th hour of open migration test.

We further analyzed the protrusion tips during rh TNFα-activated cell migration. As shown in Supplementary Figures S3J,K as well as in Supplementary Videos S2, S3, examination of the leading edge of migrating cells revealed rh TNFα-dependent formation of spindle-shaped leader cells with an aggressive phenotype oriented toward the direction of the movement. Analysis of trajectories of leader cells during the migration of TNFα-activated pHCAECs into the open field showed a statistically significant increase in Euclidean distance (from 374.4 ± 89.41 to 477.1 ± 120.4 μm, *p* = 0.0004, respectively, for control and TNFα-activated cells; Figure 2G), accumulated distances (from 554.1 ± 94.78 to 785.1 ± 138.2 μm, *p* < 0.0001, respectively, for control and TNFα-activated cells; Figure 2H), and velocity (from 0.3946 ± 0.07763 to 0.5703 ± 0.07103 μm/min, *p* < 0.0001, respectively, for control and TNFα-activated cells; Figure 2I), as compared to the control. TNFα-induced increase in the migratory potential of pHCAECs was also confirmed by their migration toward the chemo-attractant environment through 3-μm pores (Figure 2J). As shown in Supplementary Figure S3L, we observed a three-times-higher number of migrating pHCAECs after treatment with 100 ng/ml rh TNFα, when compared to the control (6.667 ± 3.240 *vs.* 22.89 ± 5.183, *p* < 0.0001). We further investigated if rh TNFα had any effect on pHCAEC formation of tube-like structures on Matrigel (Figure 2K and Supplementary Video S4). We observed a statistically significant increase in the number of tubules formed by rh TNFα-treated pHCAECs during the experiment (from 97.78 ± 10.33 to 141.3 ± 14.75 after 3 h, *p* < 0.0001; from 86.56 ± 5.833 to 100.2 ± 5.403 after 6 h, *p* < 0.0001; and from 45.22 ± 2.386 to 54.22 ± 1.856 after 6 h, *p* < 0.0001, respectively, for control and TNFα-activated cells; Supplementary Figure S3M).

In summary, these data suggest that in contrast to cooperative migration of control pHCAECs, rh TNFα activates the potential of pHCAECs to collective and coordinated invasion into a new environment through the acquisition of an aggressive phenotype characterized by spindle-like morphology oriented toward the direction of the movement. This ability was remarkably reduced due to contact inhibition between cells when pHCAECs were cultured at a high density in the closed system. The way of migration pattern of control pHCAECs was cooperative.

### Tumor Necrosis Factor α Induces Reorganization of the F-Actin Pattern in Primary Human Coronary Artery Endothelial Cells, Leading to the Formation of Aggressive Phenotypes and Reorganization of Cell–Cell Junctions

**FIGURE 3:**
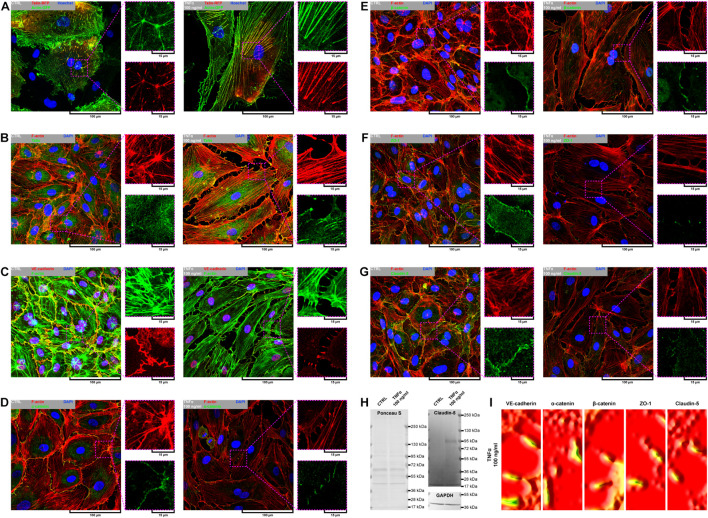
Effect of TNFα on organizational pattern of F-actin, distribution of adhesion sites, and organization of cell–cell junctions in pHCAECs. **(A)** Representative confocal micrograph of intravital organization of F-actin and focal adhesion sites. Actin-GFP (red) and talin-RFP (green) were expressed using baculoviruses. Nuclei (blue) were stained with Hoechst 33342. CTRL refers to the control (left panel), TNFα 100 ng/ml to cells treated with rh TNFα (right panel). **(B)** Representative confocal micrograph of fluorescently stained F-actin and talin. Triple fluorescent staining for talin (green), F-actin (red), and DNA (blue) was performed after fixation. CTRL refers to the control (left panel), TNFα 100 ng/ml to cells treated with rh TNFα (right panel). **(C)** Representative confocal micrograph of fluorescently stained F-actin and VE-cadherin. Triple fluorescent staining for VE-cadherin (red), F-actin (green), and DNA (blue) was performed after fixation. CTRL refers to the control (left panel), TNFα 100 ng/ml to cells treated with rh TNFα (right panel). **(D)** Representative confocal micrograph of fluorescently stained F-actin and α-catenin. Triple fluorescent staining for α-catenin (green), F-actin (red), and DNA (blue) was performed after fixation. CTRL refers to the control (left panel), TNFα 100 ng/ml to cells treated with rh TNFα (right panel). **(E)** Representative confocal micrograph of fluorescently stained F-actin and β-catenin. Triple fluorescent staining for β-catenin (green), F-actin (red), and DNA (blue) was performed after fixation. CTRL refers to the control (left panel), TNFα 100 ng/ml to cells treated with rh TNFα (right panel). **(F)** Representative confocal micrograph of fluorescently stained F-actin and ZO-1. Triple fluorescent staining for ZO-1 (green), F-actin (red), and DNA (blue) was performed after fixation. CTRL refers to the control (left panel), TNFα 100 ng/ml to cells treated with rh TNFα (right panel), ZO-1 to zonula occludens-1. **(G)** Representative confocal micrograph of fluorescently stained F-actin and claudin-5. Triple fluorescent staining for claudin-5 (green), F-actin (red), and DNA (blue) was performed after fixation. CTRL refers to the control (left panel), TNFα 100 ng/ml to cells treated with rh TNFα (right panel). **(H)** Representative western blots for claudin-5 (upper right panel) and GAPDH (lower right panel) in lysates form pHCAECs. Protein transfer efficiency was assessed by Ponceau S staining (left panel). CTRL refers to the control (left lines), TNFα 100 ng/ml to cells treated with rh TNFα (right lines). **(I)** Representative surface plots of fluorescence intensity of VE-cadherin, α-catenin, β-catenin, ZO-1, and claudin-5 from regions of cell–cell interactions between rh TNFα pHCAECs. TNFα 100 ng/ml refers to cells treated with rh TNFα, ZO-1 to zonula occludens-1.

Due to remarkable changes in the morphology of rh TNFα-activated pHCAECs and different patterns of their movement, we analyzed the organization of F-actin, distribution of focal adhesion sites, and cell–cell junction proteins. As shown in confocal micrographs in Figures 3A–G, exposition of pHCAECs to 100 ng/ml rh TNFα induced changes in the organizational pattern of F-actin. We observed the transformation of F-actin from the star-like configuration in control pHCAECs into linear stress fibers in rh TNF-activated cells, as well as changes in the organization of focal adhesion sites (Figures 3A,B and Supplementary Video S5). Analyses of the localization of actin-GFP and talin-RFP, as well as fluorescently labeled F-actin and talin, revealed that developed prominent stress fibers in rh TNFα-activated pHCAECs were preceded by the formation of tension forces between star-like arranged F-actin bundles within individual cells, which seems to determine the aggressive phenotype of pHCAECs. As shown in Figures 3A,B and Supplementary Video S5, this was related with increased cell–ECM adhesion, whereby adhesion sites were localized along newly formed and parallel-organized F-actin stress fibers (Figures 3A,B). The increased adhesion of rh TNFα-activated pHCAECs was confirmed in a wash assay (Supplementary Figures S4A,B). We found a statistically significant increase in the relative number of adherent cells per view after 1 h (from 1.033 ± 0.2395 to 1.153 ± 0.2252, *p* = 0.0052, respectively, for control and TNFα-activated cells), 2 h (from 0.9978 ± 0.2185 to 1.134 ± 0.3150, *p* = 0.0378), and 4 h (from 1.033 ± 0.2504 to 1.163 ± 0.3138, *p* = 0.0225) after seeding in complete vascular growth medium supplemented with rh TNFα (Supplementary Figures S4C–F).

Furthermore, we sought to determine whether seeding pHCAECs on different ECM proteins could affect the alignment pattern of stress fibers in response to rh TNFα. Stress fiber organization did not differ depending on ECM protein coatings and was the same as in pHCAECs cultured with rh TNFα on an uncoated surface. Indeed, actin stress fibers were parallel to each other, running along the longer axis of rh TNFα-activated pHCAECs regardless of whether they were cultured on fibronectin (Supplementary Figures S5A,B), laminin (Supplementary Figures S5C,D), collagen I (Supplementary Figures S5E,F), and collagen IV (Supplementary Figures S5G,H). It was also noticed that the formation of F-actin stress fibers was associated with increased membrane ruffling (Supplementary Video S5) and formation of punctae cell–cell junctions (Figures 3C–G). Furthermore, the discontinuous cell–cell contact areas in rh TNFα-activated pHCAECs were characterized by bright fluorescence of AJ proteins, such as VE-cadherin, α-catenin, and β-catenin (Figures 3C–E), and TJ proteins, namely, zonula occludens-1 (ZO-1) and claudin-5 (Figures 3F,G). Although we noticed posttranslational downregulation of claudin-5 after exposition of cells to rh TNFα, we also showed a strong 95-kDa reactive band, suggesting the oligomerization of claudin-5 (Figure 3H) and its different tethering of scaffold protein and F-actin due to different mechanical coupling of adjacent cells and their coordinated movement. Direct participation of AJ and TJ proteins in signalization between rh TNFα-activated cells is presented in the form of surface plots in Figure 3I.

**FIGURE 4:**
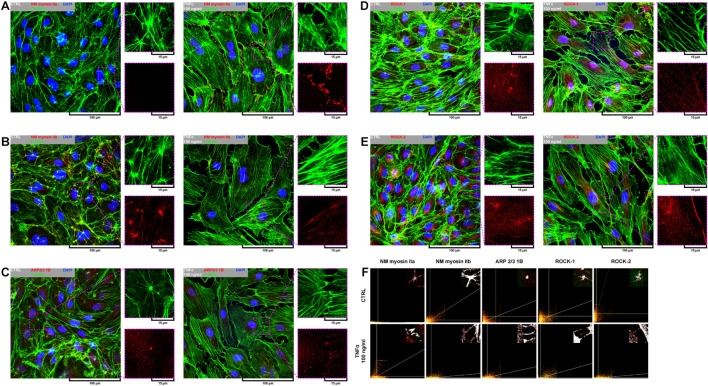
Effect of TNFα on organizational pattern of F-actin and distribution of proteins regulating actin assembly and mechanics in pHCAECs. **(A)** Representative confocal micrograph of fluorescently stained F-actin and NM myosin IIa. Triple fluorescent staining for NM myosin IIa (red), F-actin (green), and DNA (blue) was performed after fixation. CTRL refers to the control (left panel), TNFα 100 ng/ml to cells treated with rh TNFα (right panel), NM to non-muscle. **(B)** Representative confocal micrograph of fluorescently stained F-actin and NM myosin IIb. Triple fluorescent staining for NM myosin IIb (red), F-actin (green), and DNA (blue) was performed after fixation. CTRL refers to the control (left panel), TNFα 100 ng/ml to cells treated with rh TNFα (right panel), NM to non-muscle. **(C)** Representative confocal micrograph of fluorescently stained F-actin and ARP2/3. Triple fluorescent staining for subunit 1B of ARP2/3 (red), F-actin (green), and DNA (blue) was performed after fixation. CTRL refers to the control (left panel), TNFα 100 ng/ml to cells treated with rh TNFα (right panel). **(D)** Representative confocal micrograph of fluorescently stained F-actin and ROCK-1. Triple fluorescent staining for ROCK-1 (red), F-actin (green), and DNA (blue) was performed after fixation. CTRL refers to the control (left panel), TNFα 100 ng/ml to cells treated with rh TNFα (right panel). **(E)** Representative confocal micrograph of fluorescently stained F-actin and ROCK-2. Triple fluorescent staining for ROCK-2 (red), F-actin (green), and DNA (blue) was performed after fixation. CTRL refers to the control (left panel), TNFα 100 ng/ml to cells treated with rh TNFα (right panel). **(F)** Representative co-localization maps of NM myosin IIa, NM myosin IIb, subunit 1B of ARP2/3, ROCK-1, and ROCK-2 with star-like-shaped F-actin (upper panels) or F-actin of cell–cell interactions (lower panels). CTRL refers to the control (upper panels), TNFα 100 ng/ml to cells treated with rh TNFα (lower panel).

Due to a fundamental role of non-muscle (NM) myosin II, actin-related protein (ARP) 2/3, and rho-associated coiled-coil containing protein kinases 1 and 2 (ROCK-1 and ROCK-2) in processes that require cellular reshaping and movement (Bhadriraju et al., 2007; Vicente-Manzanares et al., 2009; Smith et al., 2013) and our observations of the effect of rh TNFα on F-actin rearrangement, we analyzed the localization of these proteins in both star-like-shaped F-actin bundles and rh TNFα-induced cortical F-actin stress fibers or sites of punctae cell–cell junctions. As shown in the left panel of Figures 4A–E, NM myosin IIb, ARP 2/3 1B, and ROCK-1 were localized in star-like-shaped F-actin structures, indicating their role in the cooperative transmission of tensions between adjacent cells. Interestingly, TNFα-activated cells expressed NM myosin IIa, ARP 2/3 1B, and ROCK-1 in the regions of punctae cell–cell junctions (right panel in Figures 4A–E). These observations were confirmed by the analysis of the colocalization with F-actin (Figure 4F) and determined the tensional character of discontinuous junctions between rh TNFα-activated pHCAECs.

In summary, these data, at least partially, explain the change in the migration pattern of pHCAECs in response to rh TNFα (from cooperative in the control into coordinated in rh TNFα-activated cells). We suggest that the star-like-shaped organization of F-actin bundles determines the propensity of pHCAECs to cooperative migration, important in effective contribution of ‘seal the gaps’ function, whereas rh TNFα-induced formation of prominent, parallel stress fibers and subsequent organization of punctae, but strong cell–cell junctions, allows for the directed and parallel motion of the cells during coordinated migration.

### Tumor Necrosis Factor α Induces Formation of Migrasomes Involved in Cell–Cell Signalization Between Migrating Primary Human Coronary Artery Endothelial Cells

**FIGURE 5:**
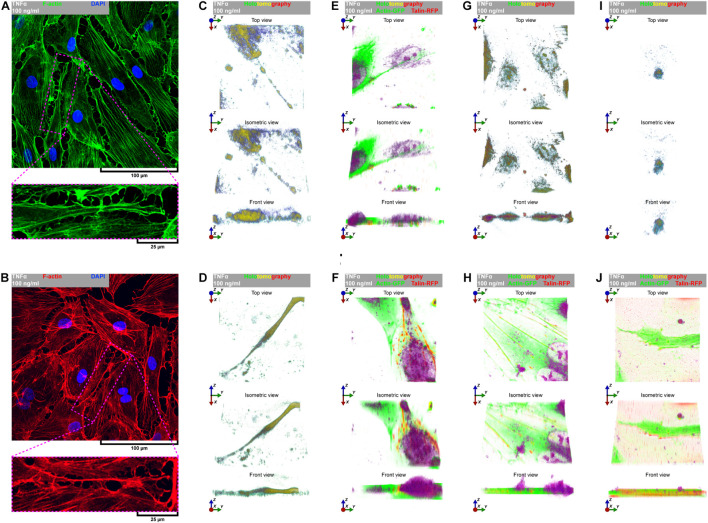
TNFα-induced formation of migrasomes and migracytosis. **(A)** Representative confocal micrograph of fluorescently stained F-actin. Double fluorescent staining for F-actin (green) and DNA (blue) was performed after fixation. TNFα 100 ng/ml to cells treated with rh TNFα. **(B)** Representative confocal micrograph of fluorescently stained F-actin. Double fluorescent staining for F-actin (red) and DNA (blue) was performed after fixation. TNFα 100 ng/ml to cells treated with rh TNFα. **(C,D,G,I)** Representative intravital holotomography micrographs of pHCAECs forming and releasing migrasomes. TNFα 100 ng/ml to cells treated with rh TNFα. **(E,F,H,J)** Representative intravital correlative holotomographic and fluorescence micrographs of pHCAECs forming and releasing migrasomes. Actin-GFP (red) and talin-RFP (green) were expressed using baculoviruses. TNFα 100 ng/ml to cells treated with rh TNFα.

Directional cell migration requires the series of changes in the structure and function of the cell at its different regions. These cover the formation of membrane-bounded cellular extensions at the leading edge and consequent retraction of the rear edge of the cell. It is widely accepted that tail retraction precedes and induces changes in migration direction and serves to maintain directionality of the force-generating leading edge of the cell (Xue et al., 2010; Theisen et al., 2012). During rh TNFα-induced coordinated migration of pHCAECs, we observed intensified formation of retraction fibers behind cells (Figures 5A–D). As shown Figures 5A–E and Supplementary Video S5, these long and tubular structures were rich in F-actin, and their formation resulted in increased adhesion and interactions between cells at the rear of migrating cells. We demonstrated that the tips of retraction fibers were continuously or discontinuously connected to the neighboring cells and were characterized by augmented adhesion, suggesting their role in transmission of F-actin-based mechanical forces for proper polarization of adjacent cells and coordination of their migration. These forces were generated by rapid contraction of retraction fibers toward the direction of cell movement (Figure 5F and Supplementary Video S5). We also frequently observed formation of migrasomes at tips or the course of retraction fibers and their release by breaking the retraction fibers (Supplementary Video S5). Moreover, as shown in Figures 5G–J, the released migrasomes tended to occur at points of cell–cell and cell–ECM contact. We also noticed that during retraction fiber breakage, some migrasomes may be released to the cell culture medium, where they seem to play a role in intercellular signalization promoting directed cell migration (Supplementary Video S6).

In summary, these data show possible ways of signalization between TNFα-activated pHCAECs during their migration. The first concerns the generation of mechanical forces during contraction of retraction fibers, and the other one is related to migracytosis. We suggest here that the formation and release of migrasomes are highly related to increased adhesion and strength of punctae or continuous intercellular junctions at the tips of retraction fibers. Moreover, our observations allow us to assume that migrasomes released by TNFα-activated pHCAECs are involved in signalization between migrating pHCAECs.

### Activation of Endogenous Expression of *TPM1* Inhibits Tumor Necrosis Factor α-Induced Inflammatory Response of Primary Human Coronary Artery Endothelial Cells and Force-Dependent Opening of Their Cell–Cell Junctions

**FIGURE 6:**
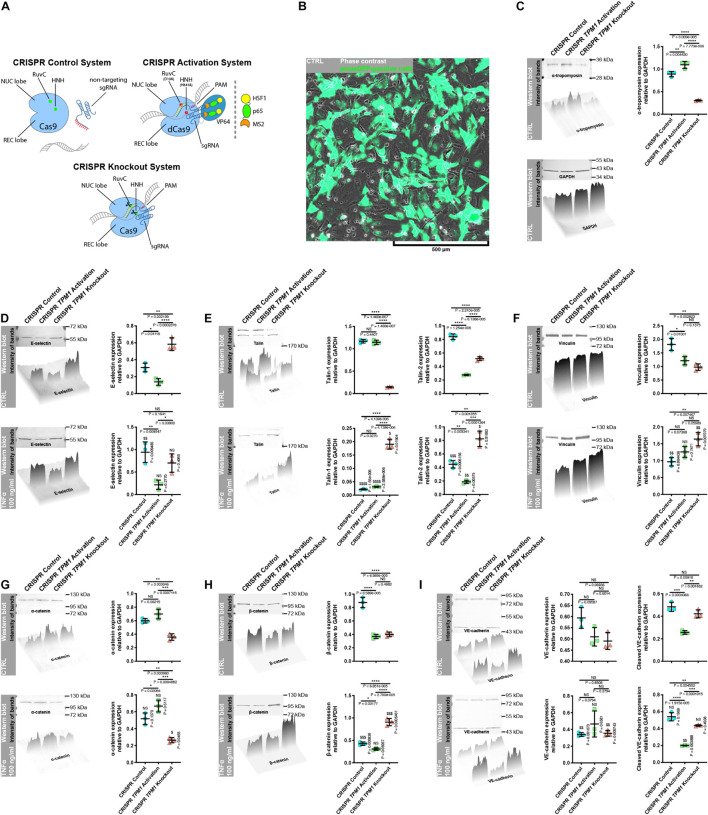
Effect of TNFα on posttranslational expression of cell–cell and cell–ECM junctional proteins in pHCAECs with CRISPR-based modulated expression of *TPM1*. **(A)** Schematic overview of CRISPR systems used in regulation of *TPM1* expression in pHCAECs. The cells were nucleofected with a CRISPR control plasmid encoding the Cas9 nuclease and a non-specific guide RNA (upper left panel), CRISPR *TPM1* activation system encoding the nuclease-deficient dCas9 fused to the transactivation domain VP64, a plasmid encoding the MS2-p65-HSF1 fusion protein, and a guide RNA targeting sequences upstream of the *TPM1* transcriptional start site (upper left panel) or CRISPR *TPM1*-knockout system encoding the Cas9 nuclease and a *TPM1*-specific 20-nt guide RNA (lower panel). **(B)** Representative micrograph of intravital detection of pmaxGFP plasmid product (green fluorescence) in nucleofected pHCAECs (contrast phase). **(C)** Representative western blots, surface plot, and densitometric analysis for α-tropomyosin (upper panel) and GAPDH (lower panel). CTRL refers to the control, ** to *p* < 0.01, **** to *p* < 0.0001. **(D)** Representative western blots, surface plot, and densitometric analysis for E-selectin. CTRL refers to the control (upper panel), TNFα 100 ng/ml to cells treated with rh TNFα (lower panel), NS to non-significant, * to *p* < 0.05, ** to *p* < 0.01, **** *p* < 0.0001, as determined by the Kruskal–Wallis test, $$ to *p* < 0.01, as determined by unpaired *t*-test. **(E)** Representative western blots, surface plot, and densitometric analysis for talin. CTRL refers to the control (upper panel), TNFα 100 ng/ml to cells treated with rh TNFα (lower panel), NS to non-significant, ** to *p* < 0.01, *** to *p* < 0.001, **** to *p* < 0.0001, as determined by the Kruskal–Wallis test, $$ to *p* < 0.01, $$$ to *p* < 0.001, $$$$ *p* < 0.0001, as determined by unpaired *t*-test. **(F)** Representative western blots, surface plot, and densitometric analysis for vinculin. CTRL refers to the control (upper panel), TNFα 100 ng/ml to cells treated with rh TNFα (lower panel), NS to non-significant, * to *p* < 0.05, ** to *p* < 0.01, as determined by the Kruskal–Wallis test, $$ to *p* < 0.01, as determined by unpaired *t*-test. **(G)** Representative western blots, surface plot, and densitometric analysis for α-catenin. CTRL refers to the control (upper panel), TNFα 100 ng/ml to cells treated with rh TNFα (lower panel), NS to non-significant, * to *p* < 0.05, ** to *p* < 0.01, *** to *p* < 0.001, as determined by the Kruskal–Wallis test, $ to *p* < 0.05, as determined by unpaired *t*-test. **(H)** Representative western blots, surface plot, and densitometric analysis for β-catenin. CTRL refers to the control (upper panel), TNFα 100 ng/ml to cells treated with rh TNFα (lower panel), NS to non-significant, * to *p* < 0.05, **** to *p* < 0.0001, as determined by the Kruskal–Wallis test, $$$ to *p* < 0.001, as determined by unpaired *t*-test. **(I)** Representative western blots, surface plot, and densitometric analysis for VE-cadherin. CTRL refers to the control (upper panel), TNFα 100 ng/ml to cells treated with rh TNFα (lower panel), NS to non-significant, ** to *p* < 0.01, *** to *p* < 0.001, **** to *p* < 0.0001, as determined by the Kruskal–Wallis test, $$ to *p* < 0.01, as determined by unpaired *t*-test.

**FIGURE 7:**
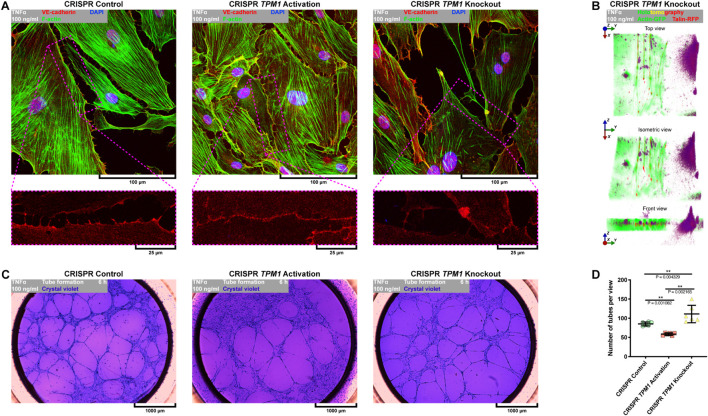
Effect of CRISPR-based regulated expression of *TPM1* on F-actin organization, continuity of cell–cell junctions, and proangiogenic prosperities of TNFα-activated pHCAECs. **(A)** Representative confocal micrographs of fluorescently stained F-actin and VE-cadherin. Triple fluorescent staining for VE-cadherin (red), F-actin (green), and DNA (blue) was performed after fixation of pHCAECs nucleofected with CRISPR control (left panel), CRISPR *TPM1* activation (middle panel), or CRISPR *TPM1*-knockout systems (right panel). TNFα 100 ng/ml refers to cells treated with rh TNFα (right panel). **(B)** Representative intravital correlative holotomographic and fluorescence micrographs of pHCAECs nucleofected with the CRISPR *TPM1*-knockout system. Actin-GFP (red) and talin-RFP (green) were expressed using baculoviruses. TNFα 100 ng/ml to cells treated with rh TNFα. **(C)** Representative bright-field micrographs presenting tube formation by pHCAECs nucleofected with CRISPR control (left panel), CRISPR *TPM1* activation (middle panel), and CRISPR *TPM1*-knockout systems cultured on Matrigel. The tubular structures were stained with crystal violet after 6 h from seeding. TNFα 100 ng/ml refers to cells treated with rh TNFα. **(D)** Number of tubes per microscopic field formed by TNFα-activated pHCAECs with CRISPR-based regulated expression of *TPM1*. ** refers to *p* < 0.01, as determined by the Kruskal–Wallis test.

It was shown by Tremblay et al. (2006) and in Figure 1 or Supplementary Figure S1 that E-selectin is the trigger of transendothelial migration but also accumulates quickly at punctae cell–cell junctions of activated endothelial cells, where it co-localizes with F-actin (Tremblay et al., 2006). We have previously shown that stabilization of F-actin by overexpression of α-tropomyosin protects endothelial integrity against L-homocysteine and cigarette smoke extract in EA.hy926 (Gagat et al., 2013, 2014). Similarly, our further studies revealed that overexpression of α-tropomyosin preserves transformed alveolar epithelial cell–cell junctions against disintegration induced by cigarette smoke extract (Gagat et al., 2016). Here, we investigated the effect of CRISPR-based regulation of endogenous *TPM1* expression on the activation and posttranslational expression of cell–cell and cell–ECM adhesion proteins. First, we activated or deactivated endogenous expression of *TPM1* using CRISPR *TPM1* activation or knockout systems introduced to the cells by the nucleofection technique (Figures 6A,B). As shown in Figure 6C, the CRISPR systems worked effectively on modulating α-tropomyosin expression in pHCAECs (from 0.888 ± 0.0578 to 1.093 ± 0.0781, *p* = 0.0044 and to 0.305 ± 0.0122, *p* < 0.0001, respectively, for CRISPR *TPM1* activation and knockout systems). Furthermore, we showed that activation of endogenous expression of *TPM1* itself downregulated posttranslational expression of E-selectin (from 0.308 ± 0.0515, *p* = 0.0112, and 0.584 ± 0.0753, *p* = 0.0002, to 0.138 ± 0.0409, respectively, as compared to the cells transfected with CRISPR control and knockout systems and inhibited rh TNFα-induced activation of pHCAECs (Figure 6D). Next, we investigated the effect of endogenous expression of *TPM1* on posttranslational expression of talin. Due to its essential role in mediating cell adhesion, most of the studies regarding talin have focused mainly on talin-1. However, talin-2 is required for the generation of traction force and formation of invadopodia (Qi et al., 2016). We observed that activation of endogenous *TPM1* expression itself downregulated posttranslational expression of talin-2 (from 0.840 ± 0.0421 to 0.274 ± 0.0066, *p* < 0.0001), whereas *TPM1* knockdown led to a downregulation of both talin-1 (from 1.169 ± 0.0417 to 0.146 ± 0.0082, *p* < 0.0001) and talin-2 (0.840 ± 0.0421 to 0.516 ± 0.0293, *p* < 0.0001). After rh TNFα treatment, we also noticed decreased expression of talin-2 (from 0.274 ± 0.0066 to 0.188 ± 0.0239, *p* = 0.0037) in *TPM1* upregulated cells and its increase in *TPM1*-knockdown pHCAECs (from 0.516 ± 0.0293 to 0.824 ± 0.1143, *p* = 0.0106. We did not observe expression of talin-1 in rh TNFα-activated pHCAECs transfected with CRISPR control and *TPM1* activation systems (Figure 6E). Talin-2 has been shown to be able to recruit vinculin in the absence of mechanical force (Austen et al., 2015). It has also been suggested that vinculin coordinates polarized cell motility and plays a central role in the regulation of endothelial barrier function via dynamic balance between centripetal forces generated by contraction of stress fibers attached to focal adhesion sites and tethering forces applied by intracellular complexes (Birukova et al., 2016). Interestingly, here, we showed similar, and independent of rh TNFα treatment, posttranslational levels of vinculin in pHCAECs with activated *TPM1* expression. Differently, we noticed rh TNFα-induced upregulation of vinculin in cells transfected with the CRISPR *TPM1*-knockout system (from 0.973 ± 0.1228 to 1.637 ± 0.1992, *p* = 0.0080; Figure 6F). Both α-catenin and vinculin cooperatively support the strength of intercellular adhesion via a mechanoresponsive link between the cadherin-β–catenin complexes and F-actin (Thomas et al., 2013). Vinculin also protects VE-cadherin-containing AJs from opening during their force-induced remodeling (Huveneers et al., 2012). The rh TNFα-independent and stable levels of vinculin in pHCAECs with activated *TPM1* expression were supported by the lack of rh TNFα-induced changes in α-catenin and β-catenin levels (Figures 6G,H). Furthermore, this was related with the rh TNFα-independent expression of VE-cadherin and decreased level of cleaved VE-cadherin (from 0.257 ± 0.0163 to 0.200 ± 0.0031, *p* = 0.0039) (Figure 6I), suggesting that activation of endogenous expression of *TPM1* leads to stabilization of AJs. Our further experiments showed that pHCAECs with activated transcription of *TPM1* were able to form continuous AJs in the presence of 100 ng/ml rh TNFα. In contrast, rh TNFα induced punctae appearance of AJs between the cells transfected with both CRISPR control and *TPM1*-knockout systems (Figure 7A). We also observed massive migracytosis after inflammatory activation of *TPM1*-knockdown pHCAECs with 100 ng/ml rh TNFα (Figures 7A,B). As shown in Figure 7C, force-dependent disorganization of cell–cell junctions was accompanied by the ability to form tubules on Matrigel. We observed a statistically significant increase in the number of tubules formed by rh TNFα-activated pHCAECs with normal and downregulated expressions of *TPM1*, as compared to the cells with activated expression of *TPM1* (from 58.50 ± 3.391 to 85.17 ± 5.076, *p* = 0.0011, and 111.0 ± 22.70, *p* = 0.0022, respectively, for the cells transfected with CRISPR *TPM1* activation, control, and *TPM1*-knockdown systems; Figure 7D).

In summary, these data suggest that activation of endogenous expression of *TPM1* inhibits inflammatory response of pHCAECs and downstream leads to the stabilization of cell–cell junctions through reducing the cleavage of VE-cadherin and maintaining stable levels of α- and β-catenins. Differently, CRISPR-based knockout of *TPM1* leads to increased migracytosis in rh TNFα-activated pHCAECs, which was confirmed by the increased angiogenic capacity of these cells in parallel with the formation of aggressive phenotypes. The migrative potential was also confirmed by the TNFα-dependent increase in posttranslational expression of talin-2, vinculin, and β-catenin.

### Activation of Endogenous Expression of *TPM1* Inhibits Tumor Necrosis Factor α-Induced Activation, Proliferation, and Migration of Primary Human Coronary Artery Smooth Muscle Cells

**FIGURE 8:**
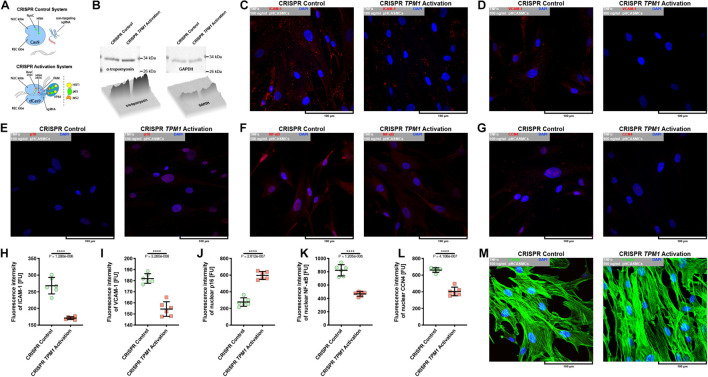
Effect of CRISPR-based activation of endogenous *TPM1* expression on expression of molecular markers of TNFα-stimulated pHCASMC migration and proliferation. **(A)** Schematic overview of CRISPR systems used in regulation of *TPM1* expression in pHCASMCs. The cells were nucleofected with CRISPR control plasmid encoding the Cas9 nuclease and a non-specific guide RNA (upper panel) or CRISPR *TPM1* activation system encoding the nuclease-deficient dCas9 fused to the transactivation domain VP64, a plasmid encoding the MS2-p65-HSF1 fusion protein, and a guide RNA targeting sequences upstream of *TPM1* transcriptional start site (lower panel). **(B)** Representative western blots and surface plots for α-tropomyosin (left panel) and GAPDH (right panel) in lysates form pHCAECs. **(C)** Representative confocal micrographs of fluorescently stained ICAM-1. Double fluorescent staining for ICAM-1 (red) and DNA (blue) was performed after fixation of TNFα-stimulated pHCASMCs nucleofected with CRISPR control (left panel) or CRISPR *TPM1* activation systems (right panel). TNFα 100 ng/ml to cells treated with rh TNFα. **(D)** Representative confocal micrographs of fluorescently stained VCAM-1. Double fluorescent staining for VCAM-1 (red) and DNA (blue) was performed after fixation of TNFα-stimulated pHCASMCs nucleofected with CRISPR control (left panel) or CRISPR *TPM1* activation systems (right panel). TNFα 100 ng/ml refers to cells treated with rh TNFα. **(E)** Representative confocal micrographs of fluorescently stained p16. Double fluorescent staining for p16 (red) and DNA (blue) was performed after fixation of TNFα-stimulated pHCASMCs nucleofected with CRISPR control (left panel) or CRISPR *TPM1* activation systems (right panel). TNFα 100 ng/ml refers to cells treated with rh TNFα. **(F)** Representative confocal micrographs of fluorescently stained NFκB. Double fluorescent staining for NFκB (red) and DNA (blue) was performed after fixation of TNFα-stimulated pHCASMCs nucleofected with CRISPR control (left panel) or CRISPR *TPM1* activation systems (right panel). TNFα 100 ng/ml refers to cells treated with rh TNFα. **(G)** Representative confocal micrographs of fluorescently stained CCN4. Double fluorescent staining for CCN4 (red) and DNA (blue) was performed after fixation of TNFα-stimulated pHCASMCs nucleofected with CRISPR control (left panel) or CRISPR *TPM1* activation systems (right panel). TNFα 100 ng/ml refers to cells treated with rh TNFα. **(H)** Fluorescence intensity of ICAM-1 measured in TNFα-stimulated pHCASMCs nucleofected with CRISPR control or CRISPR *TPM1* activation systems. **** refers to *p* < 0.0001 as determined by unpaired *t*-test. **(I)** Fluorescence intensity of VCAM-1 measured in TNFα-stimulated pHCASMCs nucleofected with CRISPR control or CRISPR *TPM1* activation systems. **** refers to *p* < 0.0001 as determined by unpaired *t*-test. **(J)** Fluorescence intensity of p16 measured in nuclei of TNFα-stimulated pHCASMCs nucleofected with CRISPR control or CRISPR *TPM1* activation systems. **** refers to *p* < 0.0001 as determined by unpaired *t*-test. **(K)** Fluorescence intensity of NFκB measured in nuclei of TNFα-stimulated pHCASMCs nucleofected with CRISPR control or CRISPR *TPM1* activation systems. **** refers to *p* < 0.0001 as determined by unpaired *t*-test. **(L)** Fluorescence intensity of CCN4 measured in nuclei of TNFα-stimulated pHCASMCs nucleofected with CRISPR control or CRISPR *TPM1* activation systems. **** refers to *p* < 0.0001 as determined by unpaired *t*-test. **(M)** Representative confocal micrographs of fluorescently stained F-actin. Double fluorescent staining for F-actin (green) and DNA (blue) was performed after fixation of TNFα-stimulated pHCASMCs nucleofected with CRISPR control (left panel) or CRISPR *TPM1* activation systems (right panel). TNFα 100 ng/ml refers to cells treated with rh TNFα.

Because CRISPR-based activation of *TPM1* expression resulted in the stabilization of interactions between pHCAECs treated with 100 ng/ml rh TNFα, we decided to evaluate whether the elevated level of α-tropomyosin was able to affect pHCASMC response to rh TNFα. As shown in Figures 8A,B, transfection of pHCASMCs with CRISPR *TPM1* activation system effectively upregulated expression of α-tropomyosin in these cells. It has been shown that expression of both ICAM-1 and VCAM-1 on intimal and medial SMCs is prominent in fibrous plaques and advanced atherosclerotic lesions and that expression of VCAM-1 correlates with intimal neovessels and mononuclear cell infiltration (Kasper et al., 1996; O’Brien et al., 1996). Here, we observed that CRISPR-based activation of *TPM1* inhibited the inflammatory response of pHCASMCs to 100 ng/ml rh TNFα, as evidenced by reduced fluorescence of ICAM-1 and VCAM-1 (both *p* < 0.0001; Figures 8C,D,H,I). Furthermore, we investigated the effect of rh TNFα on functional nuclear markers of proliferation and migration of SMCs. We observed increased levels of nuclear fluorescence for p16 (*p* < 0.0001; Figures 8E,J) and reduced fluorescence for nuclear NFκB and CCN4 (both *p* < 0.0001) in TNFα-activated pHCASMCs with upregulated expression of α-tropomyosin, as compared to TNFα-activated cells transfected with the CRISPR control system (Figures 8F,G,K,L). These observations were confirmed by the analysis of F-actin organization. As shown in Figure 8M, TNFα promoted migrative distribution of the F-actin pattern in cells with normal expression of *TPM1*, whereas in pHCASMCs with CRISPR-based activation, expression of *TPM1* supported a typical smooth muscle ‘hill and valley’ morphology.

In summary, these data suggest that activation of the endogenous expression of *TPM1* inhibits inflammatory response of pHCASMCs and leads to the inhibition of their proliferation and migration.

## Discussion

The basis of inflammation is the changes in blood vessels allowing the recruitment of leukocytes to the site of damage. It results in vessel dilatation and an increase in their permeability (Libby, 2002). Inflammation is also an essential factor accompanying both the angiogenic and atherogenic pathways (Silvestre et al., 2008). The entire inflammatory process is mediated through cytokines. TNFα is one of the representatives of proinflammatory cytokines produced mainly by monocytes or monocyte-derived macrophages and affects many processes associated with the growth and characteristics of endothelial, smooth muscle, or immune system cells (Heller and Krönke, 1994; Peppel et al., 2005). A dual role of TNFα has been shown in the angiogenic response of endothelial cells: a proangiogenic effect *in vivo* and an antiangiogenic *in vitro* (Fràter-Schröder et al., 1987; Sainson et al., 2008). It has been also suggested that TNFα inhibits endothelial cell proliferation *in vitro* (Fràter-Schröder et al., 1987). The TNFα apoptotic response of human umbilical vein and aortic and coronary artery endothelial cells when cultured *in vitro* was also shown (Fràter-Schröder et al., 1987; Chen et al., 2004; Rastogi et al., 2012; Jiang et al., 2016). The reason for that can be a host organism used for the production of recombinant TNFα. *Escherichia coli* is one of the organisms of choice for the production of recombinant proteins, such as TNFα, commonly used in vascular biology studies. However, protein expression in this system leads to many problems such as inclusion body formation related to incorrect disulfide bond formation, improper folding, or reduction in biological activity due to incomplete folding or mutations in cDNA (Rosano and Ceccarelli, 2014; Tavallaei et al., 2015). In the present study, we used rh TNFα produced in HEK293 cells, which effectively activated functional expression of E-selectin and VCAM-1 in pHCAECs. Although classical endothelial cell activation is defined by a shift in the expression of E-selectin, VCAM-1, and ICAM-1 (Gimbrone et al., 1997), we observed only a weak induction of functional expression of ICAM-1 (data not shown). Wu et al. (2004) indicated the heterogeneity of pHCAECs’ response to TNFα stimulation, e.g., due to pathologic conditions of human coronary artery donors (Wu et al., 2004). However, it has been shown that soluble markers of endothelial injury are not uniformly increased in patients with documented coronary artery disease and that the plasma level of ICAM-1 did not allow identification of endothelial injury in such patients (Semaan et al., 2000). It was also suggested that the deficiency of ICAM-1 either alone or in combination with the deficiency of VCAM-1 did not alter nascent lesion formation, indicating the importance of VCAM-1 in the initiation of atherosclerosis (Cybulsky et al., 2001). As the activation of endothelium is associated with enhanced interactions with leukocytes (Woodfin et al., 2009), we confirmed the flattening, firm adhesion, and further migration of Jurkat T cells on the surface of the rh TNFα-activated pHCAECs. These data are consistent with the results received by Jaczewska et al. (2014) who demonstrated increased interactions between Jurkat T cells and HUVECs following treatment with INF-γ or TNFα. In the course of the inflammatory process, the capture of leukocytes on the surface of the endothelium is possible due to the interaction of very late antigen-4 integrin on the leukocyte with selectins and VCAM-1 in endothelial cell walls (Abdala-Valencia et al., 2011). Similarly, the observed phenomenon of stronger adhesion of lymphocytes to endothelial monolayer was associated with increased expression of E-selectin and VCAM-1 cell adhesion molecules, which was also confirmed by Lu et al. (2016) and Munro et al. (1989). It was also suggested that the activation of endothelium includes not only changes in levels of adhesion molecules expression but also their redistribution from cell junctions to non-junctional membranes (JAM-A, JAM-C, and PECAM-1) or internalization from the plasma membrane (VE-cadherin) (Reglero-Real et al., 2016). Additionally, the effect of TNFα on TEM seems to depend on stimulation time. Within shorter stimulation times, leukocytes bind preferentially to the junctional regions of endothelial cells, whereas within longer periods, receptors in the junctional region are no longer easily available and TEM is intensified by cytoskeletal rearrangement and increased endothelial permeability (Jaczewska et al., 2014). Our observations seem to confirm this assumption since cytoskeletal rearrangement leading to the formation of discontinuous cell–cell junctions and intercellular gaps was finished at about 12–16 h from rh TNFα treatment.

**FIGURE 9:**
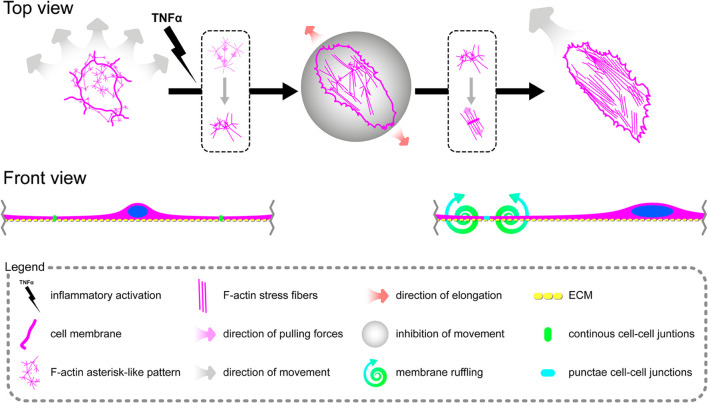
Schematic overview of TNFα-induced reorganization of F-actin and its effect on migration of pHCAECs.

Activation of pHCAECs following rh TNFα treatment led to morphological changes. One of the most obvious was a transformation of the shape of the cells into a spindle-like one and oriented toward the direction of cell migration. Similar to that found in our study, TNFα-induced spindle-shaped, narrowed, and elongated morphology was observed in HUVECs and hCMEC/D3 cells and correlated with the increased paracellular permeability (Miyazaki et al., 2017; Ni et al., 2017). Here, we showed that rh TNFα-induced change of the pHCAEC shape was related to the coordinated rearrangement of actin cytoskeleton from the star-like-shaped F-actin bundles into prominent, parallel-organized stress fibers. This perfectly explains the transition of polygonal cobblestone-like pHCAECs to a uniformly spindle-shaped and aligned monolayer. Aster- or star-like-shaped structures were described by Fritzsche et al. (2017) as self-organized F-actin patterns, achieved by polarity sorting of actin filaments. In the *in silico* analysis, they showed two main nucleation pathways of this actin patterning. In the first scenario, Arp2/3 complexes bind to preexisting F-actin and nucleate new filaments from their pointed ends (−), leaving the barbed ends (+) pointing outward. In the second one, myosin II crosslinks with F-actin at their barbed ends (+) at the pattern centers, resulting in the point ends (−) pointing outward (Fritzsche et al., 2017). In our study, the core of star-like-shaped F-actin structures co-localized with ARP2/3, whereas radiating bundles co-localized with NM myosin IIb and ROCK-1. Furthermore, the analysis of the trajectories of cells showed that the star-like-shaped organization of F-actin bundles determines the propensity of pHCAECs to cooperative migration in any direction, important in the effective contribution of barrier function. Differently, parallel-organized stress fibers in activated pHCAECs promoted directed and parallel motion during the coordinated migration (Figure 9). These results suggest that rh TNFα induces the formation of the aggressive angiogenic phenotype of pHCAECs, which was confirmed in various migratory experiments.

The transformation of the F-actin pattern induced by rh TNFα was closely related to abnormalities in junctional regions and the distribution of focal adhesion sites. We proved that rh TNFα promotes discontinuous cell–cell contact, preceded by membrane ruffling during polarization of pHCAECs to achieve a spindle-like shape promoting their migration. Interestingly, TNFα-activated cells expressed NM myosin IIa, ARP 2/3 1B, and ROCK-1 in the regions of punctae cell–cell junctions. It was shown that NM myosin IIa controls cadherin clustering at AJs in a Rho-dependent manner, allowing proper adhesion of epithelial cells (de Beco et al., 2012). Smutny et al. (2010) showed that NM myosin IIa promotes the accumulation of E-cadherin in the AJs while NM myosin IIB stabilizes the associated perijunctional actin ring, increasing cell–cell adhesion and preventing them from disruptive forces. However, Efimova and Svitkina (2018) showed an association of NM myosin IIa with contractile actin bundle running parallel to linear AJs in endothelial cells. In our study, the NM myosin IIa correlated with a bright fluorescence of AJ and TJ proteins in the regions of punctae cell–cell junctions. Furthermore, we showed oligomerization of claudin-5 in activated pHCAECs. Claudin-5 preferentially forms hexamers, which make cell contacts much stronger than in the case of the monomeric form (Krause et al., 2008). Considering the above, this suggests that rh TNFα-induced punctae intercellular junctions are strong and play an important role in directed cell migration of pHCAECs, allowing follower cells to trail the leaders (Ozawa et al., 2020).

**FIGURE 10:**
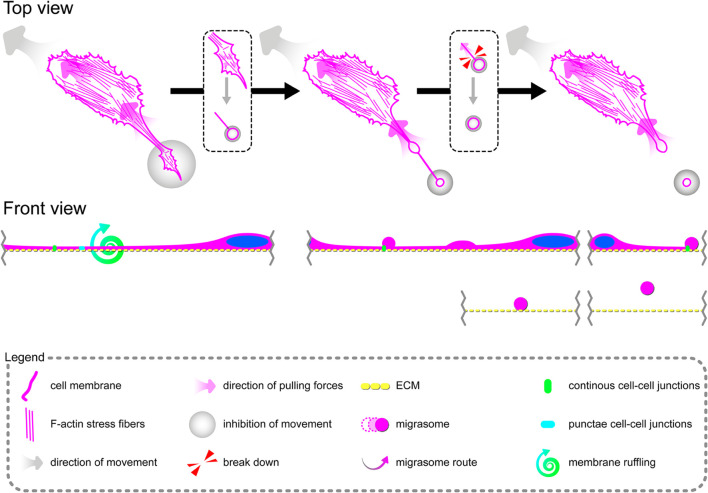
Schematic overview of TNFα-induced formation of migrasomes and migracytosis in pHCAECs.

**FIGURE 11:**
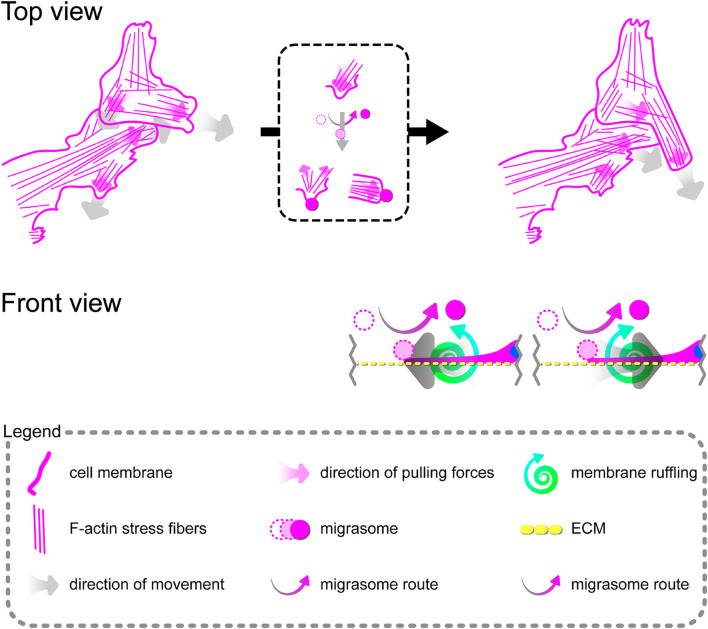
Schematic overview of the mechanism of pHCAEC response to their interaction with migrasomes.

Ma et al. (2015) identified and described extracellular membrane-bounded vesicular structures that are characteristically generated along retraction fibers in migrating cells. They named these pomegranate-like structures as migrasomes and showed their formation in various cell lines, including MEF (mouse embryonic fibroblast), NIH3T3 (mouse embryonic fibroblast), HaCaT (human keratinocyte), MDA-MB-231 (human breast cancer), HCT116 (human colon cancer), SW480 (human adenocarcinoma), MGC803 (human gastric carcinoma), SKOV-3 (human ovarian adenocarcinoma), and B16 (mouse melanoma), and organs, such as the eye, lung, and intestine. These structures have been also observed in the lumen of blood vessels or pulmonary alveoli (Ma et al., 2015). However, the mechanism of their formation and biological or clinical importance is still unknown. Huang et al. (2019) proposed the mechanism of migrasome growing as an assembly of tetraspanin- and cholesterol-enriched membrane microdomains into micron-scale macrodomains. It has been also shown that tetraspanins are localized at digitation junctions, which reflect the transition processes before the establishment or after the disassembly of stable cell–cell junctions (Huang et al., 2018). Here, we showed intensified formation of migrasomes in TNFα-stimulated pHCAECs and that their formation is highly dependent on cell–cell and cell–ECM interaction, suggesting their role in the transmission of F-actin-based mechanical forces for proper polarization of adjacent cells and coordination of the cell migration direction. Furthermore, we frequently observed their release by breaking the retraction fibers, which resulted in their stay at points of cell–cell and/or cell–ECM contact as well as their release to the cell culture medium (Figure 10). Finally, we showed that floating migrasomes exert local cytoskeletal rearrangement and motility response (Figure 11), suggesting their involvement in intercellular signalization promoting directed migration of activated pHCAECs.

The data presented here showed that rh TNFα induces F-actin reorganization resulting in mechanical disruption of cell–cell junction continuity through the promotion of directed migration of pHCAECs. In this case, methods for regulation of the actin cytoskeleton structure might be clinically potent. One of such targets may be α-tropomyosin, which belongs to a family of actin-binding proteins. Tropomyosins are coiled-coil parallel dimers that form a head-to-tail polymer along the length of actin filaments regulating their access of other actin-binding proteins (Gunning et al., 2015). In mammals, over 40 tropomyosin isoforms are produced by alternative promoter selection, alternative splicing and/or 3′ end processing of four different genes: *TPM1*, *TPM2*, *TPM3*, and *TPM4* (Denz et al., 2004). Tropomyosin variants are classified into two major groups: high molecular weight (HMW; ∼284 amino acids and molecular weight between 33 and 40 kDa) and low molecular weight (LMW; ∼247 amino acids and molecular weight between 28 and 34 kDa) (Schevzov et al., 2011). In muscle cells, all actin filaments are saturated with tropomyosins and regulate muscle contraction in a calcium-dependent manner, while in non-muscle cells, saturation of actin vary from 30% to 90%, depending on the cell type and tropomyosins stabilize the actin cytoskeleton and participate in many cellular processes, including motility, cell–cell adhesion, and cell–extracellular interactions (Perry, 2001; Humayun-Zakaria et al., 2019; Janco et al., 2019). Our previous studies have shown that increased expression of α-tropomyosin protects endothelial integrity against L-homocysteine and cigarette smoke extract in EA.hy926 (Gagat et al., 2013, 2014) and preserves transformed alveolar epithelial cell–cell junctions against disintegration induced by cigarette smoke extract (Gagat et al., 2016). α-Tropomyosin was also indicated to function as a tumor suppressor primarily by inhibiting cell proliferation, angiogenesis, and metastasis in renal cell carcinoma (Wang et al., 2019). Here, we showed that CRISPR-based activation of the endogenous expression of *TPM1* inhibits the inflammatory response of pHCAECs to TNFα and leads to the stabilization of cell–cell junctions through reduced cleavage of VE-cadherin and the maintenance of the stable levels of α- and β-catenins. We also showed that CRISPR-based knockout of *TPM1* leads to an increased angiogenic capacity of rh TNFα-activated pHCAECs and augments the formation of migrasomes and migracytosis in these cells. These findings pushed us to question how pHCASMCs with the activated expression of *TPM1* will respond to rh TNFα. Wang et al. (2011) showed that *TPM1* is a validated target of microRNA-21, which negatively regulates its posttranslational expression and leads to arteriosclerosis obliterans. They also showed that overexpression of *TPM1* decreased proliferation and migration of hASMCs, whereas its silencing significantly attenuated the antiproliferative and anti-migratory roles of the miR-21 inhibitor (Wang et al., 2011). Our results indicate that CRISPR-based activation of *TPM1* expression results in inhibition of the inflammatory response of pHCASMCs and exerts antiproliferative and anti-migratory activity.

Our study has several limitations. First of all, the study was performed in static conditions. However, the effect of rh TNFα is similar to that in *in vivo* studies, showing the proangiogenic action of TNFα. Also, due to the study design, we did not perform migratory tests on different ECM proteins, but we showed a lack of differences in the organization of F-actin in rh TNFα-activated pHCAECs when cultured on fibronectin, laminin, collagen I, and collagen IV coatings. Proliferation and migration of TNFα-activated pHCASMCs were also assessed based on the nuclear localization and fluorescence intensity of p16, NFκB, and CCN4. Furthermore, our study is based on morphological and semiquantitative analyses, but it allows for fast and easy translation of the findings into applied clinical studies. Finally, we used commercially available pHCAECs and pHCASMCs. Although we know the cause of the death of cell donors, we cannot unequivocally exclude that there was no generalized shock reaction of unknown duration that could affect the studied cells. In order to exclude this possible and reversible effect, only cells within three to four passages were used for all the experiments. It is also worth remembering that *TPM1* gene products occur in at least 29 isoforms (Cooley and Bergtrom, 2001). Unfortunately, in our study, we were unable to identify the isoform targeted by the applied CRISPR systems. Identification of the specific isoform, the expression of which determines the observed effect, is extremely important in the context of subsequent studies and their clinical application. However, in our study, tropomyosin was detected using the F-6 antibody (Santa Cruz) specific for an epitope mapping between amino acids 123 and 161. Furthermore, the molecular weight of the detected isoform of tropomyosin was in the range 33–35 kDa in pHCAECs and 33–34 kDa in pHCASMCs.

In conclusion, the present investigations demonstrated that rh TNFα-induced activation of pHCAECs results in actin cytoskeleton reorganization, promoting their directed and coordinated migration. We also proposed that the formation and release of migrasomes are highly related to increased adhesion and junctional strength of tips of retraction fibers with adjacent cells and that they play a role in intercellular signalization promoting directed cell migration. Furthermore, we showed that stabilization of F-actin through the activation of endogenous expression of *TPM1* inhibits inflammatory response of pHCAECs, allowing formation of continuous cell–cell junctions, and exerts antiproliferative and anti-migratory effects in pHCASMCs. Additional *in vivo* studies are needed to gain a better understanding of the role of α-tropomyosin in atherosclerosis and angiogenesis, as well as to examine the potential of *TPM1* as a candidate therapy target for proinflammatory vascular disorders. However, our findings may be adapted during the design and development of new coronary stent devices.

## Data Availability Statement

The original contributions presented in the study are included in the article/Supplementary Material, further inquiries can be directed to the corresponding author.

## Author Contributions

MG and AG conceptualized the project and designed the experiments. MG, WZ, KM, and AK performed the experiments. MG and DG interpreted the results. MG, KM, and WZ performed image analysis. MG and WZ performed statistical analysis. MG and WZ drafted the manuscript. KM and DG edited the manuscript. AK-W and JZ critically reviewed the manuscript. MG gained funding for the study. AG provided supervision. All authors contributed to the article and approved the submitted version.

## Conflict of Interest

MG, DG, and AG are the authors on a patent disclosing the intravascular stent, especially for coronary vessels which inner covering comprises monoclonal anti-VE-cadherin antibodies and a system of induction of tropomyosin-1 expression. The remaining authors declare that the research was conducted in the absence of any commercial or financial relationships that could be construed as a potential conflict of interest.

## Publisher’s Note

All claims expressed in this article are solely those of the authors and do not necessarily represent those of their affiliated organizations, or those of the publisher, the editors and the reviewers. Any product that may be evaluated in this article, or claim that may be made by its manufacturer, is not guaranteed or endorsed by the publisher.

## Supplementary Material

Supplementary Figure S1 TNFα-induced expression of E-selectin in pHCAECs. Triple fluorescent staining for E-selectin (green), F-actin (red), and DNA (blue) was performed after fixation. CTRL refers to the control (top panel), TNFα 100 ng/ml to cells treated with rh TNFα.

Supplementary Figure S2 TNFα-induced expression of VCAM-1 in pHCAECs. **(A)** Representative micrographs of intravital detection of VCAM-1 using magnetic beads coated with anti-VCAM-1 monoclonal antibodies (bright field). F-actin (red) and nuclei (blue) were stained fluorescently after fixation. TNFα 100 ng/ml refers to cells treated with rh TNFα, mAb to monoclonal antibodies. **(B)** Representative confocal micrograph of fluorescently stained VCAM-1. Triple fluorescent staining for VCAM-1 (green), F-actin (red), and DNA (blue) was performed after fixation. TNFα 100 ng/ml refers to cells treated with rh TNFα. **(C)** Representative micrographs of adherent Jurkat T cells (contrast phase and fluorescence) after 30 min of their co-culture with pHCAECs (contrast phase). Nuclei of Jurkat cells (blue) were stained with Hoechst 33342. CTRL refers to the control (top panel), TNFα 100 ng/ml to cells treated with rh TNFα (bottom panel).

Supplementary Figure S3 Effect of TNFα on migration of pHCAECs. **(A)** Rose plot presenting direction bias of pHCAEC migration in confluence within 36 h. CTRL refers to the control (left panel), TNFα 100 ng/ml to cells treated with rh TNFα (right panel), ^∗∗^ to *p* < 0.01, ^****^ to *p* < 0.0001, as determined by Rayleigh test. **(B)** Euclidean distance of pHCAECs during 36-h migration in 100% confluence. CTRL refers to the control, TNFα 100 ng/ml to cells treated with rh TNFα, NS to non-significant to the control, as determined by unpaired *t*-test. **(C)** Accumulated distance of pHCAECs during 36-h migration in 100% confluence. CTRL refers to the control, TNFα 100 ng/ml to cells treated with rh TNFα, ^****^ to *p* < 0.0001, as determined by unpaired *t*-test. **(D)** Velocity of pHCAECs during 36-h migration in 100% confluence. CTRL refers to the control, TNFα 100 ng/ml to cells treated with rh TNFα, ^****^ to *p* < 0.0001, as determined by unpaired *t*-test. **(E)** Correlation between Euclidean distance of pHCAECs and time during 36-h migration in 100% confluence. CTRL refers to the control, TNFα 100 ng/ml to cells treated with rh TNFα, NS to non-significant, ^∗∗^ to *p* < 0.01, as determined by Pearson’s correlation coefficient analysis. **(F)** Correlation between accumulated distance of pHCAECs and time during 36-h migration in 100% confluence. CTRL refers to the control, TNFα 100 ng/ml to cells treated with rh TNFα, NS to non-significant, ^∗∗^ to *p* < 0.01, as determined by Pearson’s correlation coefficient analysis. **(G)** Correlation between velocity of pHCAECs and time during 36-h migration in 100% confluence. CTRL refers to the control, TNFα 100 ng/ml to cells treated with rh TNFα, NS to non-significant, ^∗∗^ to *p* < 0.01, as determined by Pearson’s correlation coefficient analysis. **(H)** Correlation between time, accumulated distance, Euclidean distance or velocity, and directness of control pHCAECs during 36-h migration in 100% confluence. CTRL refers to the control, NS to non-significant, ^****^ to *p* < 0.0001. **(I)** Correlation between time, accumulated distance, Euclidean distance or velocity, and directness of TNFα pHCAECs during 36-h migration in 100% confluence. TNFα 100 ng/ml refers to cells treated with rh TNFα, NS to non-significant, ^∗^ to *p* < 0.05, ^****^ to *p* < 0.0001. **(J)** Representative contrast phase micrograph presenting pHCAEC migration to the wound site. Curves indicate leading edge of migrating cells, arrows the direction of the movement. CTRL refers to the control (left panel), TNFα 100 ng/ml to cells treated with rh TNFα (right panel). **(K)** Representative contrast phase micrograph presenting pHCAEC migration to the open field. Curves indicate leading edge of migrating cells, arrows the direction of the movement. CTRL refers to the control (left panel), TNFα 100 ng/ml to cells treated with rh TNFα (right panel). **(L)** Number of pHCAECs migrated through 3-μm pores per microscopic field. CTRL refers to the control, TNFα 100 ng/ml to cells treated with rh TNFα, ^****^ to *p* < 0.0001, as determined by unpaired *t*-test. **(M)** Number of tubes per microscopic field formed by pHCAECs on Matrigel after 3, 6, and 12 h after seeding. CTRL refers to the control, TNFα 100 ng/ml to cells treated with rh TNFα, ^∗^ to *p* < 0.05, ^****^ to *p* < 0.0001, as determined by Kruskal–Wallis test, $$$$ to *p* < 0.0001, as determined by unpaired *t*-test.

Supplementary Figure S4 Effect of rh TNFα on adhesion of pHCAECs during initial 4 h from the seeding. **(A)** Number of adherent pHCAECs per microscopic field from the control and TNFα-activated pHCAECs. **(B)** Representative micrographs of adherent pHCAECs (contrast phase) within 4 h after seeding. CTRL refers to the control (top panel), TNFα 100 ng/ml to cells treated with rh TNFα (bottom panel). **(C)** Relative number of adherent pHCAECs per microscopic field from the control and TNFα-activated pHCAECs after 0.5 h from seeding. NS refers to non-significant to the control, as determined by unpaired *t*-test. **(D)** Relative number of adherent pHCAECs per microscopic field from the control and TNFα-activated pHCAECs after 1 h from seeding. ^∗∗^ refers to *p* < 0.01, as determined by unpaired *t*-test. **(E)** Relative number of adherent pHCAECs per microscopic field from the control and TNFα-activated pHCAECs after 2 h from seeding. ^∗^ refers to *p* < 0.05, as determined by unpaired *t*-test. **(F)** Relative number of adherent pHCAECs per microscopic field from the control and TNFα-activated pHCAECs after 2 h from seeding. ^∗^ refers to *p* < 0.05, as determined by unpaired *t*-test.

Supplementary Figure S5 Effect of rh TNFα on organizational pattern of F-actin in pHCAECs cultured on different ECM coatings. **(A)** Representative confocal micrograph of fluorescently stained F-actin in pHCAECs cultured on fibronectin. Double fluorescent staining for F-actin (green) and DNA (blue) was performed after fixation. CTRL refers to the control (left panel), TNFα 100 ng/ml to cells treated with rh TNFα (right panel). **(B)** Representative confocal micrograph of fluorescently stained F-actin in pHCAECs cultured on fibronectin. Double fluorescent staining for F-actin (red) and DNA (blue) was performed after fixation. CTRL refers to the control (left panel), TNFα 100 ng/ml to cells treated with rh TNFα (right panel). **(C)** Representative confocal micrograph of fluorescently stained F-actin in pHCAECs cultured on laminin. Double fluorescent staining for F-actin (green) and DNA (blue) was performed after fixation. CTRL refers to the control (left panel), TNFα 100 ng/ml to cells treated with rh TNFα (right panel), EHS to Engelbreth-Holm-Swarm. **(D)** Representative confocal micrograph of fluorescently stained F-actin in pHCAECs cultured on laminin. Double fluorescent staining for F-actin (red) and DNA (blue) was performed after fixation. CTRL refers to the control (left panel), TNFα 100 ng/ml to cells treated with rh TNFα (right panel), EHS to Engelbreth-Holm-Swarm. **(E)** Representative confocal micrograph of fluorescently stained F-actin in pHCAECs cultured on collagen I. Double fluorescent staining for F-actin (green) and DNA (blue) was performed after fixation. CTRL refers to the control (left panel), TNFα 100 ng/ml to cells treated with rh TNFα (right panel). **(F)** Representative confocal micrograph of fluorescently stained F-actin in pHCAECs cultured on collagen I. Double fluorescent staining for F-actin (red) and DNA (blue) was performed after fixation. CTRL refers to the control (left panel), TNFα 100 ng/ml to cells treated with rh TNFα (right panel). **(G)** Representative confocal micrograph of fluorescently stained F-actin in pHCAECs cultured on collagen IV. Double fluorescent staining for F-actin (green) and DNA (blue) was performed after fixation. CTRL refers to the control (left panel), TNFα 100 ng/ml to cells treated with rh TNFα (right panel), EHS to Engelbreth-Holm-Swarm. **(H)** Representative confocal micrograph of fluorescently stained F-actin in pHCAECs cultured on collagen IV. Double fluorescent staining for F-actin (red) and DNA (blue) was performed after fixation. CTRL refers to the control (left panel), TNFα 100 ng/ml to cells treated with rh TNFα (right panel), EHS to Engelbreth-Holm-Swarm.

Supplementary Video S1 Migration of Jurkat T cells on the surface of pHCAECs. CTRL refers to the control (upper panel), TNFα 100 ng/ml to cells treated with rh TNFα (lower panel). Nuclei of Jurkat cells were stained with Hoechst 33342. Time-lapse images were acquired for 2 min at 3.33-s intervals after 30 min from co-culture of pHCAECs (contrast phase) with counterstained Jurkat cells (contrast phase and fluorescence). Frame rate, 10 frames/s.

Supplementary Video S2 TNFα-induced changes in migration of pHCAECs to the wound site. CTRL refers to the control (upper panel), TNFα 100 ng/ml to cells treated with TNFα (lower panel). Time-lapse images were acquired for 24 h at 10-min intervals. Frame rate, 20 frames/s.

Supplementary Video S3 TNFα-induced changes in migration of pHCAECs to the open field. CTRL refers to the control (upper panel), TNFα 100 ng/ml to cells treated with TNFα (lower panel). Time-lapse images were acquired for 24 h at 10-min intervals. Frame rate, 20 frames/s.

Supplementary Video S4 TNFα-induced changes in the ability of pHCAECs to form tubes on Matrigel. CTRL refers to the control (left panel), TNFα 100 ng/ml to cells treated with rh TNFα (right panel). Time-lapse images were acquired for 24 h at 10-min intervals. Frame rate, 20 frames/s.

Supplementary Video S5 TNFα-induced changes in organization pattern of F-actin and distribution of focal adhesion sites. Actin-GFP (red) and talin-RFP (green) were expressed using baculoviruses. TNFα 100 ng/ml refers to cells treated with rh TNFα. Time-lapse images were acquired for 18 h at 10-min intervals after 2 h from treatment. Frame rate, 20 frames/s.

Supplementary Video S6 TNFα-induced formation of migrasomes and migracytosis. Actin-GFP (red) and talin-RFP (green) were expressed using baculoviruses. TNFα 100 ng/ml refers to cells treated with rh TNFα. Time-lapse images were acquired for 6.5 h at 10-min intervals after 15 h from treatment. Frame rate, 3 frames/s.

Supplementary Video S7 Interactions of pHCAECs with migrasome released to cell culture medium. Actin-GFP (green) and talin-RFP (red) were expressed using baculovirus system. TNFα 100 ng/ml refers to cells treated with rh TNFα. Time-lapse images were acquired for 1.25 h at 5-min intervals after 18 h from treatment. Frame rate, 3 frames/s.
