# Supplementary figures and images for: CRISPR-Based Activation of Endogenous Expression of TPM1 Inhibits Inflammatory Response of Primary Human Coronary Artery Endothelial and Smooth Muscle Cells Induced by Recombinant Human Tumor Necrosis Factor α
Source: Front Cell Dev Biol. 2021 Sep 17;9:668032. doi: 10.3389/fcell.2021.668032 (PMC8484921; doi:10.3389/fcell.2021.668032)

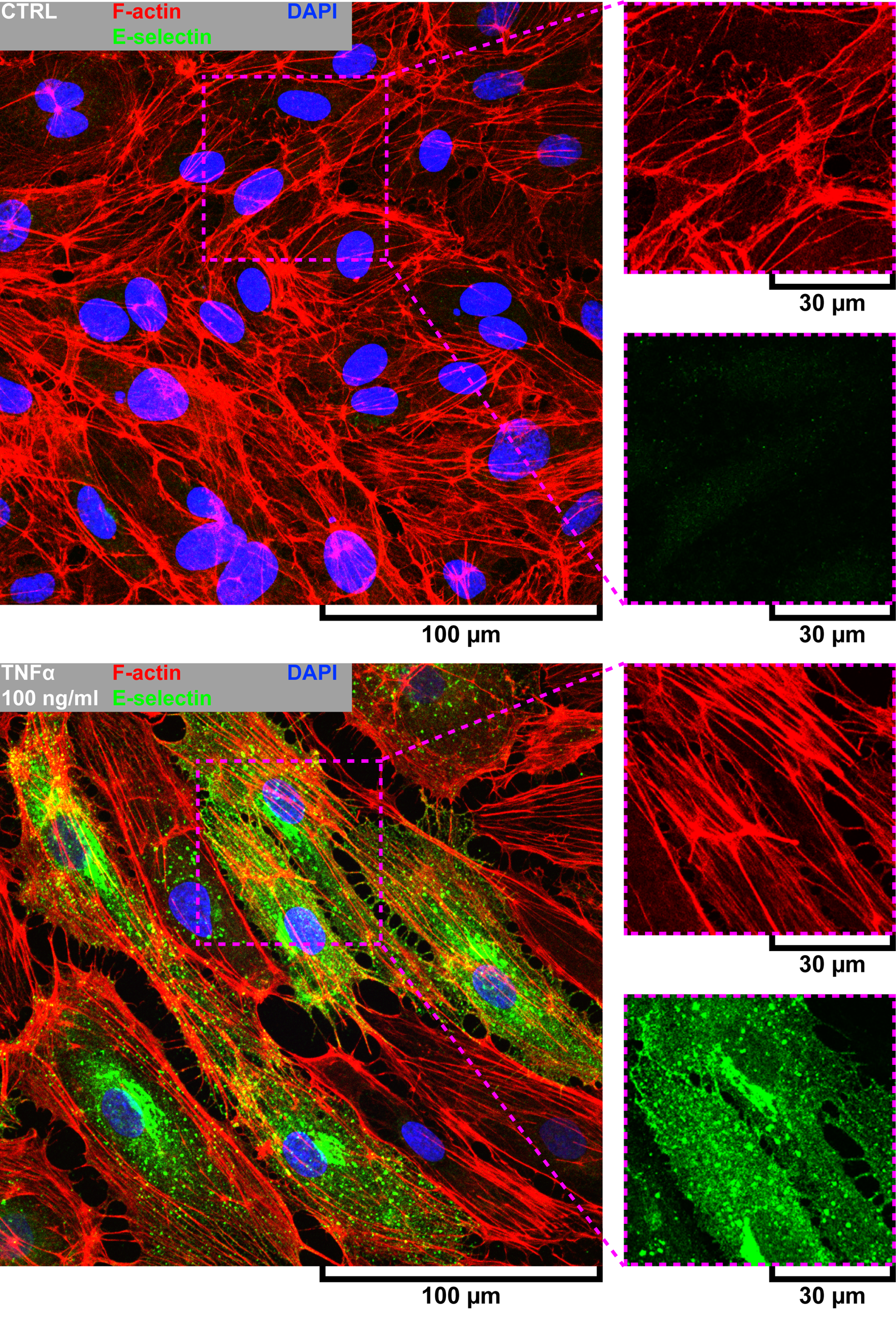

Supplement: Supplementary Figure S1 — TNFα-induced expression of E-selectin in pHCAECs. Triple fluorescent staining for E-selectin (green), F-actin (red), and DNA (blue) was performed after fixation. CTRL refers to the control (top panel), TNFα 100 ng/ml to cells treated with rh TNFα. [file Image_1.TIF]

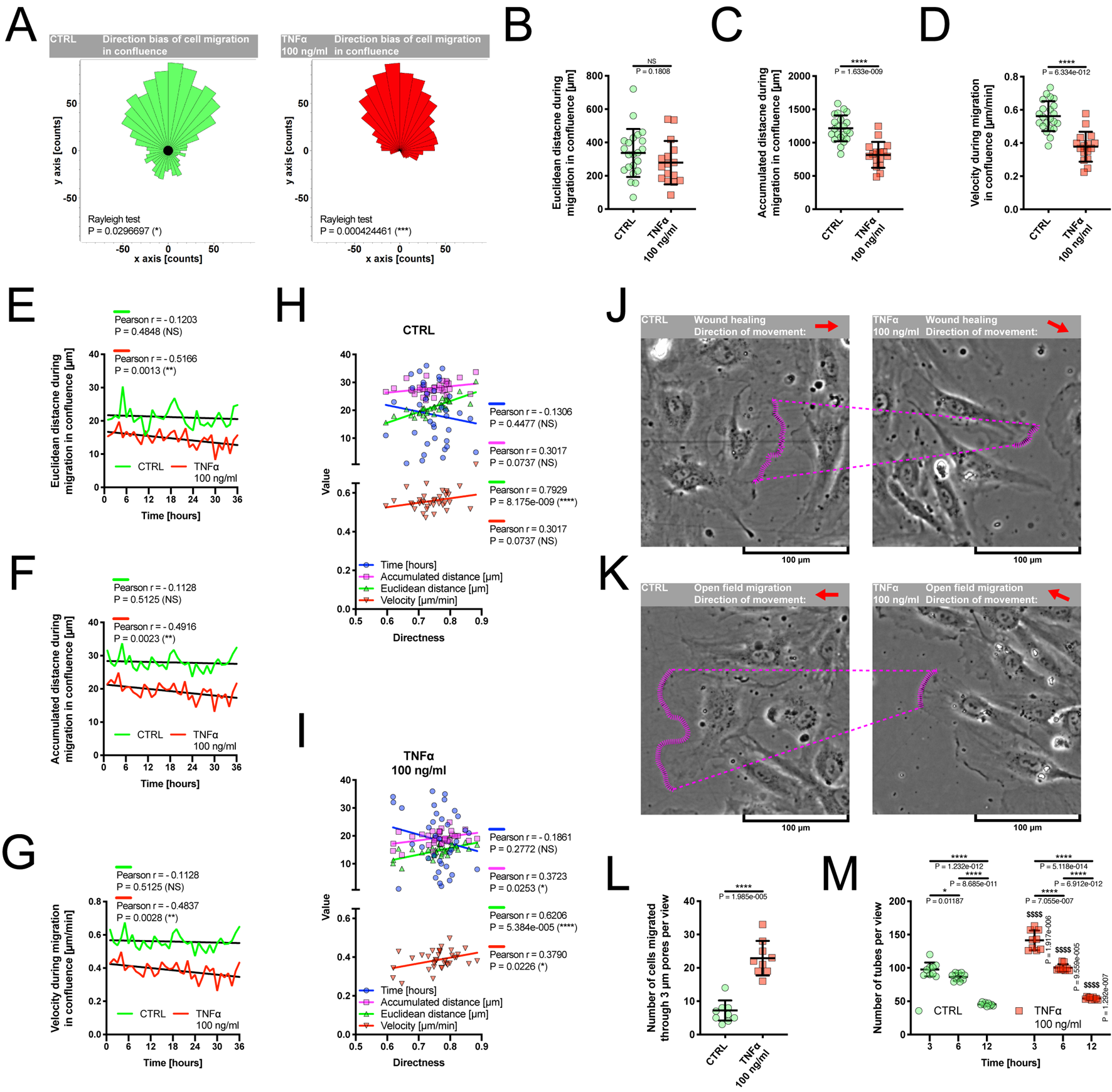

Supplement: Supplementary Figure S3 — Effect of TNFα on migration of pHCAECs. (A) Rose plot presenting direction bias of pHCAEC migration in confluence within 36 h. CTRL refers to the control (left panel), TNFα 100 ng/ml to cells treated with rh TNFα (right panel), ∗∗ to p < 0.01, **** to p < 0.0001, as determined by Rayleigh test. (B) Euclidean distance of pHCAECs during 36-h migration in 100% confluence. CTRL refers to the control, TNFα 100 ng/ml to cells treated with rh TNFα, NS to non-significant to the control, as determined by unpaired t-test. (C) Accumulated distance of pHCAECs during 36-h migration in 100% confluence. CTRL refers to the control, TNFα 100 ng/ml to cells treated with rh TNFα, **** to p < 0.0001, as determined by unpaired t-test. (D) Velocity of pHCAECs during 36-h migration in 100% confluence. CTRL refers to the control, TNFα 100 ng/ml to cells treated with rh TNFα, **** to p < 0.0001, as determined by unpaired t-test. (E) Correlation between Euclidean distance of pHCAECs and time during 36-h migration in 100% confluence. CTRL refers to the control, TNFα 100 ng/ml to cells treated with rh TNFα, NS to non-significant, ∗∗ to p < 0.01, as determined by Pearson’s correlation coefficient analysis. (F) Correlation between accumulated distance of pHCAECs and time during 36-h migration in 100% confluence. CTRL refers to the control, TNFα 100 ng/ml to cells treated with rh TNFα, NS to non-significant, ∗∗ to p < 0.01, as determined by Pearson’s correlation coefficient analysis. (G) Correlation between velocity of pHCAECs and time during 36-h migration in 100% confluence. CTRL refers to the control, TNFα 100 ng/ml to cells treated with rh TNFα, NS to non-significant, ∗∗ to p < 0.01, as determined by Pearson’s correlation coefficient analysis. (H) Correlation between time, accumulated distance, Euclidean distance or velocity, and directness of control pHCAECs during 36-h migration in 100% confluence. CTRL refers to the control, NS to non-significant, **** to p < 0.0001. (I) Correlation betwee [file Image_3.TIF]

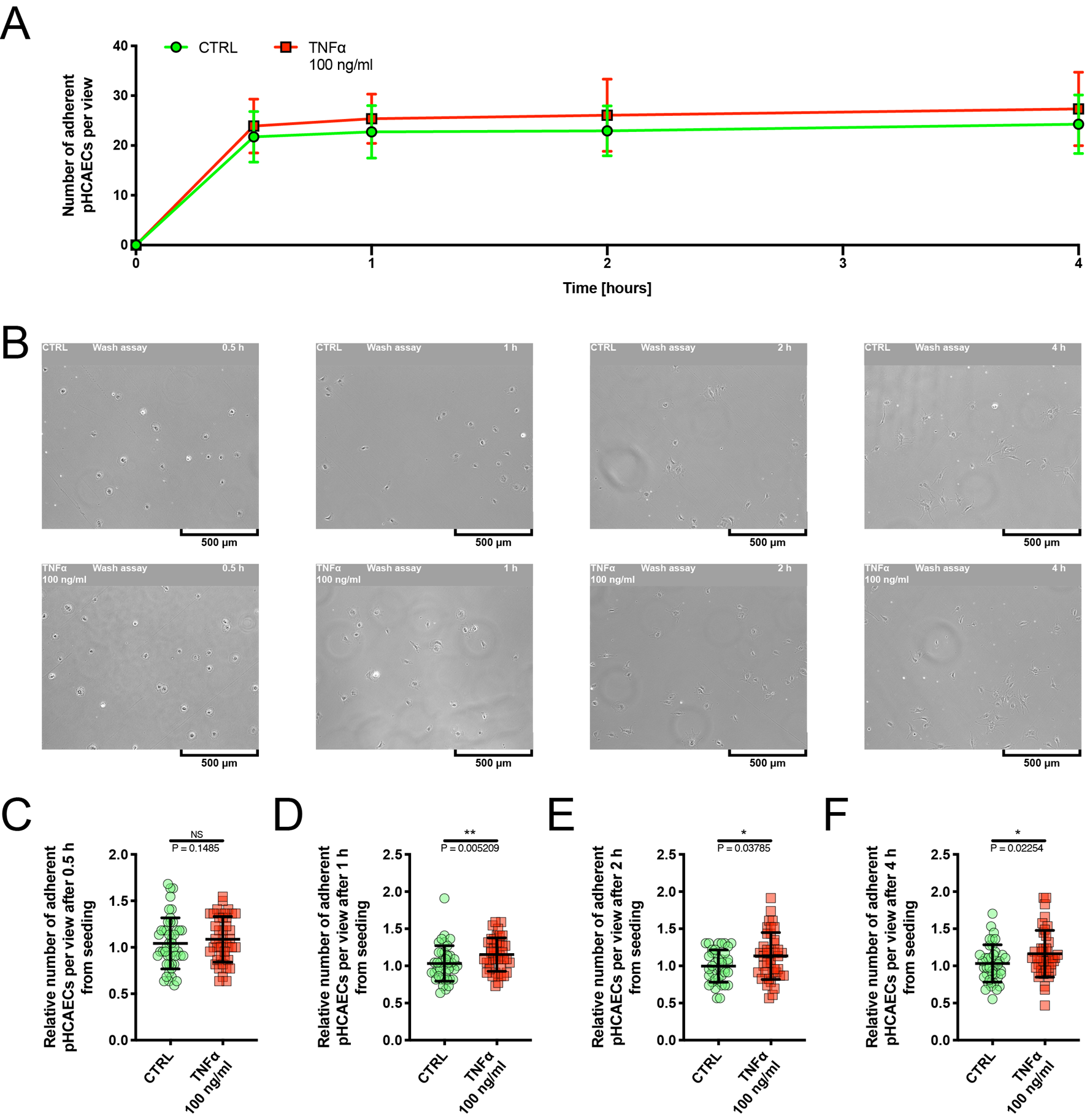

Supplement: Supplementary Figure S4 — Effect of rh TNFα on adhesion of pHCAECs during initial 4 h from the seeding. (A) Number of adherent pHCAECs per microscopic field from the control and TNFα-activated pHCAECs. (B) Representative micrographs of adherent pHCAECs (contrast phase) within 4 h after seeding. CTRL refers to the control (top panel), TNFα 100 ng/ml to cells treated with rh TNFα (bottom panel). (C) Relative number of adherent pHCAECs per microscopic field from the control and TNFα-activated pHCAECs after 0.5 h from seeding. NS refers to non-significant to the control, as determined by unpaired t-test. (D) Relative number of adherent pHCAECs per microscopic field from the control and TNFα-activated pHCAECs after 1 h from seeding. ∗∗ refers to p < 0.01, as determined by unpaired t-test. (E) Relative number of adherent pHCAECs per microscopic field from the control and TNFα-activated pHCAECs after 2 h from seeding. ∗ refers to p < 0.05, as determined by unpaired t-test. (F) Relative number of adherent pHCAECs per microscopic field from the control and TNFα-activated pHCAECs after 2 h from seeding. ∗ refers to p < 0.05, as determined by unpaired t-test. [file Image_4.TIF]

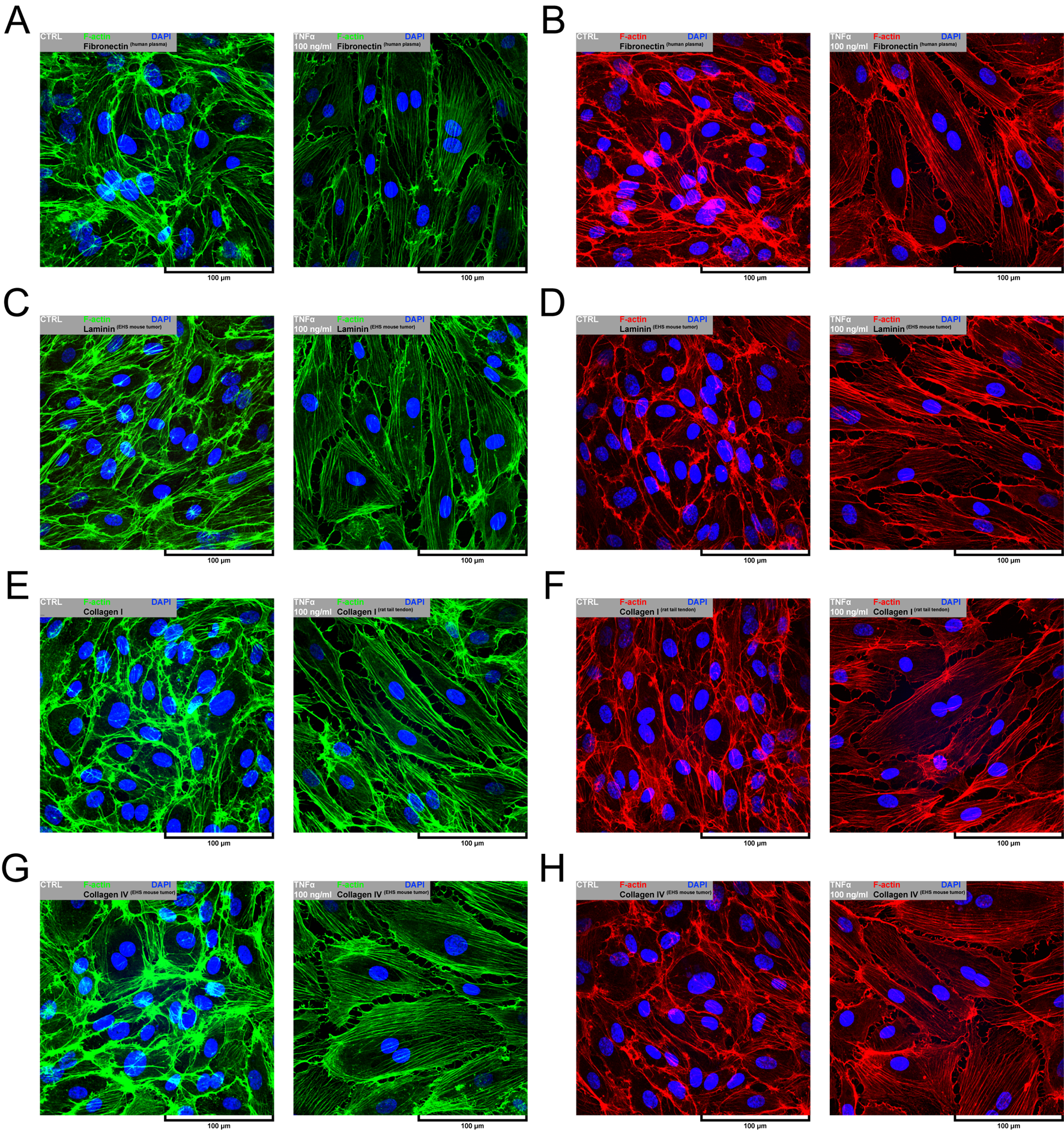

Supplement: Supplementary Figure S5 — Effect of rh TNFα on organizational pattern of F-actin in pHCAECs cultured on different ECM coatings. (A) Representative confocal micrograph of fluorescently stained F-actin in pHCAECs cultured on fibronectin. Double fluorescent staining for F-actin (green) and DNA (blue) was performed after fixation. CTRL refers to the control (left panel), TNFα 100 ng/ml to cells treated with rh TNFα (right panel). (B) Representative confocal micrograph of fluorescently stained F-actin in pHCAECs cultured on fibronectin. Double fluorescent staining for F-actin (red) and DNA (blue) was performed after fixation. CTRL refers to the control (left panel), TNFα 100 ng/ml to cells treated with rh TNFα (right panel). (C) Representative confocal micrograph of fluorescently stained F-actin in pHCAECs cultured on laminin. Double fluorescent staining for F-actin (green) and DNA (blue) was performed after fixation. CTRL refers to the control (left panel), TNFα 100 ng/ml to cells treated with rh TNFα (right panel), EHS to Engelbreth-Holm-Swarm. (D) Representative confocal micrograph of fluorescently stained F-actin in pHCAECs cultured on laminin. Double fluorescent staining for F-actin (red) and DNA (blue) was performed after fixation. CTRL refers to the control (left panel), TNFα 100 ng/ml to cells treated with rh TNFα (right panel), EHS to Engelbreth-Holm-Swarm. (E) Representative confocal micrograph of fluorescently stained F-actin in pHCAECs cultured on collagen I. Double fluorescent staining for F-actin (green) and DNA (blue) was performed after fixation. CTRL refers to the control (left panel), TNFα 100 ng/ml to cells treated with rh TNFα (right panel). (F) Representative confocal micrograph of fluorescently stained F-actin in pHCAECs cultured on collagen I. Double fluorescent staining for F-actin (red) and DNA (blue) was performed after fixation. CTRL refers to the control (left panel), TNFα 100 ng/ml to cells treated with rh TNFα (right panel). (G) Representative confocal micrograph of [file Image_5.TIF]
